# Mouse Lung Fibroblast Resistance to Fas-Mediated Apoptosis Is Dependent on the Baculoviral Inhibitor of Apoptosis Protein 4 and the Cellular FLICE-Inhibitory Protein

**DOI:** 10.3389/fphys.2017.00128

**Published:** 2017-03-14

**Authors:** Sanda A. Predescu, Jian Zhang, Cristina Bardita, Monal Patel, Varun Godbole, Dan N. Predescu

**Affiliations:** ^1^Department of Internal Medicine, Division of Pulmonary and Critical Care, Rush University, Medical CollegeChicago, IL, USA; ^2^Department of Biological Sciences, Columbia UniversityNew York, NY, USA; ^3^Northwestern University Feinberg School of MedicineChicago, IL, USA

**Keywords:** caveolin 1 and caveolin 2 knockouts, double-siRNA, Bid, caspase-8, IAPs, lung fibrosis

## Abstract

A characteristic feature of idiopathic pulmonary fibrosis (IPF) is accumulation of apoptotic resistant fibroblasts/myofibroblasts in the fibroblastic foci. As caveolin (Cav)-null mice develop pulmonary fibrosis (PF), we hypothesized that the participating fibroblasts display an apoptosis-resistant phenotype. To test this hypothesis and identify the molecular mechanisms involved we isolated lung fibroblasts from Cav-null mice and examined the expression of several inhibitors of apoptosis (IAPs), of c-FLIP, of Bcl-2 proteins and of the death receptor CD95/Fas. We found significant increase in XIAP and c-FLIP constitutive protein expression with no alteration of Bcl-2 and lower levels of CD95/Fas. The isolated fibroblasts were then treated with the CD95/Fas ligand (FasL) to induce apoptosis. While the morphological and biochemical alterations induced by FasL were similar in wild-type (wt) and Cav-null mouse lung fibroblasts, the time course and the extent of the alterations were greater in the Cav-null fibroblasts. Several salient features of Cav-null fibroblasts response such as loss of membrane potential, fragmentation of the mitochondrial continuum concurrent with caspase-8 activation, and subsequent Bid cleavage, prior to caspase-3 activation were detected. Furthermore, M30 antigen formation, phosphatidylserine expression and DNA fragmentation were caspase-3 dependent. SiRNA-mediated silencing of XIAP and c-FLIP, individually or combined, enhanced the sensitivity of lung fibroblasts to FasL-induced apoptosis. Pharmacological inhibition of Bcl-2 had no effect. Together our findings support a mechanism in which CD95/Fas engagement activates caspase-8, inducing mitochondrial apoptosis through Bid cleavage. XIAP and c-FLIP fine tune this process in a cell-type specific manner.

## Introduction

Idiopathic pulmonary fibrosis (IPF), -is a chronic lung disease-, and the most frequent and relentless form of interstitial pneumonias (Noble et al., 2012). It manifests over several years and is characterized by drastic scarring of the lung tissue and an unyielding loss of lung function in the absence of any known initiating factors (Martinez et al., 2005; King et al., 2011). IPF: (i) is a diffuse lung syndrome that belongs to a subgroup of diseases known as idiopathic interstitial pneumonia (IIP), (ii) has an unknown etiology and is associated with a pathologic pattern known as usual interstitial pneumonia (UIP), and (iii) is often referred to as IPF/UIP (Meltzer and Noble, 2008). Areas of patchy fibrosis, sub-pleural honeycombing, and fibroblastic foci (Crystal et al., 2002) composed of a collagenous matrix with embedded apoptosis-resistant fibroblasts/myofibroblasts (Selman et al., 2001) represent the histological blueprint of IPF.

Current evidence emphasizes the important role of apoptosis in normal lung homeostasis and in pathogenesis of a variety of lung diseases (Chin et al., 1998; Uhal, 2002; Demedts et al., 2006; Kuwano, 2007). Lung cells apoptosis occurs through a highly regulated proteolytic process that eliminates unwanted, damaged, or altered cells. Depending on the cell type and local cues, apoptosis could have helpful or harmful effects. It has been suggested that, apoptosis in different cellular populations is involved in the pathogenesis of IPF and the presence of unresolved IPF lesions. However, its precise role is not well-established. Understanding the factors that mediate cell-specific survival and death, becomes of paramount importance for deciphering the initiation, propagation and prognosis of IPF.

Apoptosis of the lung epithelium has been suggested to alter of the integrity of the alveolo-capillary basement membrane, a stage recognized as a point of “no return” for the fibrotic process (Wallace et al., 2007). This alteration assists the epithelial-to-mesenchymal transition of some lung cells (Tanjore et al., 2009), while failure of the re-epithelilization and re-endothelialization processes leads to the destruction of the lung architecture (Keeley et al., 2010), and the emergence of apoptosis-resistant myofibroblasts (Hecker et al., 2011; Hecker and Thannickal, 2014). In IPF the emerging myofibroblasts play important roles in tissue remodeling and fibrogenesis. Some myofibroblasts not only acquire characteristics of apoptosis-resistance but also display enhanced production of extracellular matrix components and cytokines (Chilosi et al., 2006; Kuwano, 2007; Jin and Dong, 2011; Hinz et al., 2012; Hu and Phan, 2013). It has been shown that when these modified cells are located in the fibro-proliferative areas, they display continuous activation of several signaling pathways that promote cell survival, such as the PI3K/AKT pathway (Fattman, 2008). Moreover, several mechanisms have been proposed to account for the apoptosis-resistance of fibroblasts, including: (i) upregulation of the WNT5A gene that triggers fibroblast proliferation and inhibits apoptosis through a non-canonical WNT/β-catenin pathway (Vuga et al., 2009), (ii) diminished prostaglandin E2 production caused by persistent activation of the pro-survival kinase Akt (Maher et al., 2010), (iii) cytokine-mediated pathways such as, activated TGFβ1 overexpression via the inhibition of plasminogen activation (Horowitz et al., 2008) along with inducible nitric oxide synthase activation (Zhang and Phan, 1999) and (iv) increased endothelin-1 levels via pro-survival PI3/AKT signaling (Swigris and Brown, 2010). Interestingly, low expression of cell surface FasL, vs. high levels of the apoptosis-inhibiting soluble Fas (sFas) has also been related to fibro-proliferation (Buhling et al., 2005). CD95/Fas, a death receptor of the tumor necrosis factor receptor (TNF-R) superfamily, has been implicated in the development of IPF owing to its ability to promote lung injury via apoptosis of alveolar epithelial cells (Hagimoto et al., 1997). Previous studies have shown that alveolar epithelial cells, fibroblasts, and myofibroblasts express CD95 *in vitro*, as well as in the fibrotic lung tissues of IPF patients (Kazufumi et al., 1997; Kuwano et al., 1999; Kuwano, 2007). These findings suggest a potential dual role of CD95 function. CD95 initiate epithelial apoptosis and, facilitate myofibroblasts apoptosis and clearance during the resolution phase of lung injury (Kuwano, 2007; Cha et al., 2010; Golan-Gerstl et al., 2012). The molecular events that are activated by FasL to trigger apoptosis are well-characterized (Strasser et al., 2009). The events begin with the activation of caspase-8, which after leaving the death-induced signaling complex (DISC), targets specific substrates within the cytosol, i.e., the effector caspases-3, caspase-7, and the pro-apoptotic BH3 interacting-domain death agonist protein (Bid), that are critical for CD95-mediated apoptosis (Luo et al., 1998; Stennicke et al., 1998; Kuwano, 2007). In this pathway, the levels of CD95 on the cell surface determine the extent of effector caspases activation as well as the efficiency of both; the assembly of the DISC and proteolytic activation of Bid; whereas the levels of inhibitors of these processes, such as the inhibitors of apoptotic proteins (IAPs), –particularly the X-linked inhibitor of apoptosis protein (XIAP) and FADD-like interleukin-1b-converting enzyme (FLICE) inhibitory proteins (c-FLlP; Kaufmann et al., 2012), restrict their activation.

Significantly, the recent availability of caveolin-1 (Cav1^−/−^), and caveolin-2 (Cav2^−/−^) deficient mice revealed a main phenotype of generalized fibrosis (Drab et al., 2001; Razani et al., 2001, 2002).

Caveolin-1 (cav1) has been associated with the regulation of cell signaling and endocytosis in a plethora of cell types. Yet, its association with the fibrosis remains largely uncharacterized despite the evidence that the protein has inhibitory effects on matrix proteins production and the caveolin gene was demonstrated, by knockout, to function primarily as an antifibrotic gene (Gvaramia et al., 2013). However, the Cav-null mice are not yet well-characterized and comparisons of these mice to human disease are limited (Tourkina et al., 2005, 2008; Wang et al., 2006; Kim et al., 2008). Emerging data demonstrates that in the lung tissue and fibroblasts of IPF patients, there is a reduction of Cav-1 expression relative to the control samples (Wang et al., 2006); as well as in the lung tissues of patients with skin scleroderma (Del Galdo et al., 2008; Tourkina et al., 2008; Gvaramia et al., 2013). An accumulated body of evidence indicates the presence of low levels of cav1 in human IPF, and demonstrates that cav1 is an antifibrogenic molecule. Nevertheless, one of the main functions of the cav1 chaperone–for cav2 exit to the Golgi apparatus has not, also, been considered. We recently reported that: (i) Cav2^−/−^ mice, with normal levels of cav1, develop lung fibrosis over time and (ii) cav2, not cav1 drives the fibrotic process (Predescu et al., 2011, 2012).

Based on these findings, we aimed to investigate the apoptotic cellular changes and the main molecular mechanisms involved in the response of lung fibroblasts isolated from Cav1^−/−^ to Cav 2^−/−^ mice to apoptosis inducer FasL. We investigated the molecular mechanisms and cellular manifestations used by the cav null fibroblast to attain apoptosis-resistance, by comparing them to fibroblasts from wt mice and with two other lung cell types. We analyzed the sequential morphological and biochemical changes of the apoptosis-resistant fibroblasts, and determined the role of IAPs and c-FLlP in their susceptibility to FasL- induced apoptosis.

## Materials and methods

### Materials

All tissue culture reagents were from Lonza (Walkersville, PA) and their detailed description is specified in Supplementary Data. All EM reagents and the polylysine coated glass cover slips were from EM Science (Hatfield, PA), while the JEOL JEM-1220 transmission electron microscope fitted with a Gatan digital camera, and the JEOL JSM-6320F high resolution Field Emission scanning electron microscope, from electron microscopy facility of University of Illinois at Chicago, were used for taking pictures. All chemicals and reagents for electrophoresis along with the antibodies (Abs) used and different kits used are listed in Supplementary Data.

### Animals

Cav1^−/−^, strain Cav1^tm1Mls^/J, and the control mice, strain B6129SF2/J, hereafter referred as wild type (wt) mice, were obtained from Jackson laboratory, while Cav2^−/−^, on the same background as the wt-mice were provided by Professor Michael Lisanti. The Cav2^−/−^ mice were genotyped every 3–6 months and breed to form a small Cav2-null colony. Mice were housed under standardized pathogen-free conditions in the Rush University animal facility. All experimental procedures involving mice were performed under direct supervision of Rush Institutional Animal Care and Use Committee and the surgical procedures executed under anesthesia as detailed in Supplementary Data. All efforts were made to minimize suffering; in addition all mouse studies adhered to APS's Guiding Principles in the Care and Use of Vertebrate Animals in Research and Training and were performed according with the IACUC approved protocol number 14-023.

### Cell isolation, culture, treatment, and fractionation

Lung fibroblasts from wt-, Cav 1^−/−^ and Cav 2^−/−^ mice were derived as previously described in Baglole et al. (2005).

One day post-confluent fibroblast cultures were exposed to different treatments as follows: (i) for apoptosis induction detailed conditions are given in the following entry; (ii) for XIAP activity inhibition the specific inhibitors ABT-199 or ABT-236 (dissolved in dimethyl sulfoxide as 1,000-fold concentrated stock) were used at a final concentration of 1 μm; (iii) for cellular fractionation, confluent culture were treated with Fas L for different time points, as specified in the figure legends, or were untreated (controls–c) and then a cytosolic and a membrane fraction were prepared by differential centrifugation as in Predescu et al. (2001).

Isolation and characterization of murine lung endothelial cells (ECs) was done as in Predescu et al. (2013) while murine epithelial cells (EpCs) were isolated as in Blickwede and Borlak (2005); the cells were used between passages 4–6. An extended description of cell isolation, characterization and culture is given in Supplementary Data.

### Induction of apoptosis

All experiments were performed on quiescent fibroblasts (grown in DMEM + 0.1% FCS for 24 h). Preliminary experiments carried out with different concentrations of FasL (5, 10, 30, 60, 90, 100 μg/mL) and CHM (10, 20, 40, 60, 90, 100, 120 μg/mL) allowed us to establish the cellular threshold–90 μg/mL FasL in the presence of 100 μg/mL CHM–needed for apoptosis induction, this condition will be referred through the paper as FasL-induced apoptosis. Additionally, isolated fibroblasts were treated for different time points (1, 2, 4, 8, 12, 24, and 48 h); and also we changed the succession of FasL and CHM administration in order to establish the best conditions for apoptosis induction.

### siRNA studies

For single transfections 2 × 10^6^ cells in a 400 μL volume were transfected with specific siRNAs and the working conditions listed in Supplementary Data. For double knockdowns, cells were plated on 60 cm^2^ plastic Petri dishes, rested for 24 h and the firs transfection, using lipofectamine, was performed as above. The second Silencer Select siRNA was then transfected using lipofectamine as described in Predescu et al. (2013) and as described in Supplementary Data.

### Cellular proliferation assay on monomeric and polymeric collagen

Lung fibroblasts isolated from mice were serum-starved for 24 h and placed on monomeric collagen (MC; 200 μg/mL), or on polymeric collagen (PC; 2 mg/mL) in DMEM + 10% FCS. Plates covered with two-dimensional matrices (MC) were obtained as in Xia et al. (2008) while polymeric matrices (PC) were prepared as in Tian et al. (2002). Cells (10^3^ cells/well) were grown for 48 h and then the MTT assay for cellular proliferation was performed as in Patel et al. (2012). The outcomes were read at 570 nm using an Epoch plate reader and the results are expressed as number of cells/mL.

### Measurement of CD95, fas bound to the cell surface and secreted into cell culture media

Expression level of FasL mRNA was analyzed by RT- PCR using the primers and the conditions detailed in Supplementary Data. The relative amounts of membrane cell-bound FasL were measured by western blotting (WB), of membrane fraction obtained by cell fractionation as described under cell culture and the details given in Supplementary Data. The Fas secreted into the culture media (sFas) was measured with the MFL00 kit according to the instructions, while the CD95 expressed in the membranes of isolated cells was detected by cell surface ELISA using the same Ab utilized for immunostaining.

### AnnexinV staining

Phosphatidyl serine (PS) expression on the outer leaflet of plasmalemma proper was monitored by staining with AnnexinV while propidium iodide was used to identify the necrotic cells. Only the adherent cells were evaluated by fluorescence microscopy using appropriate filters. Methodological details are given in Supplementary Data.

### TUNEL and cellular DNA fragmentation

Permeabilized cells exposed to TUNEL mixture, were processed as per manufacturer protocol, and examined with a Zeiss Axioimager M1 fluorescence microscope as detailed in Supplementary Data.

### Caspase activation

The presence of caspase-2, -3, -6, -8, and -9 activity in FasL + CHM treated fibroblasts was determined using 5 × 10^6^ cells (wt-, Cav1^−/−^, Cav2^−/−^) per sample and the protocol recommended by the manufacturer of KHZ1001-kit. All experiments were performed in triplicates and the fold-increase in caspase-2, -3, -6, -8, and -9 activities was determined by comparison to non-treated controls. After having established the presence of mentioned caspases we used 2 × 10^7^cells for every type of fibroblasts stimulated with FasL to measure the increase in specific enzymatic activities of caspase-3, -8, -9, at 24 h and 48 h, using the protocols provided by the corresponding kits, and a thorough description of the method(s) is reported in Supplementary Data.

### Mitochondrial function

The mitochondrial transmembrane potential (ΔΨm) was determined with the JC-1 assay kit as per manufacturer instructions using a 96 well black plate and 5 × 10^5^ cells as described in Supplementary Data.

The structural integrity of the mitochondrial continuum was assessed by transmission electron microscopy (TEM) of unstimulated and FasL-stimulated fibroblasts, as well as by determining its DNA copy number after FasL induced apoptosis of mouse fibroblasts using the method of Miller et al. (2003) and the conditions detailed in Supplementary Data.

### Keratin 18 fragmentation assay

The specific proteolytic cleavage of Keratin 18 (K18) is an event-taking place before disruption of membrane asymmetry and breakage of DNA strands. K18 as a type I intermediate filament protein and one of the major component of epithelial cells, is cleaved by activated caspases, exposing a neo-epitope (M30) that is specifically recognized by M30 CytoDEATH™ monoclonal Ab, which detects only apoptotic cells (not viable or necrotic cells). Consequently, the usage of M30 CytoDEATH™ Ab as a unique tool for easy and reliable determination of apoptosis from very early until well-advanced stages was rationalized to specifically recognize single cells and on tissue sections only cells undergoing apoptosis. However, accumulating data suggests its presence in human myofibroblasts in the late stages of pulmonary fibrosis (Moodley et al., 2004; Larsson, 2008; Ley et al., 2011), and therefore we have used it for immunostaining of isolated fibroblasts from wt, Cav1^−/−^ and Cav2 ^−/−^.

### Western blotting and immunofluorescence

Usually 20–80 μg total proteins per lane were separated on a SDS-PAGE minigel and transferred to nitrocellulose membranes (NC) as in Towbin et al. (1979). Strips of NC were incubated with the primary Abs and processed as in Predescu et al. (2005). The detailed conditions for WB as well as for immunofluorescence are given in Supplementary Data.

### Transmission electron microscopy (TEM) and scanning electron microscopy (SEM)

For TEM, Wt-, Cav 1^−/−^ and Cav 2^−/−^ fibroblasts were grown in 35 mm plastic Petri dishes until 70–80% confluent, exposed to FasL, then at predetermined time point, the media was removed and the adherent cells were prepared for flat embedding as in Predescu et al. (2003), while SEM examination of cultured fibroblasts was performed as in Knezevic et al. (2009) and in both cases details are provided in Supplementary Data.

### Statistical analysis

All experiments were conducted a minimum of three times. Statistical analysis using SPSS version 17.0 software were performed by one-way ANOVA for pair-wise multiple comparisons, while comparisons among groups were performed with a Neuwman-Keul multiple comparison test; *p* < 0.05 was considered significant.

## Results

### Characterization of murine lung fibroblasts isolated from Cav1^−/−^ and Cav2^−/−^ mice

**Figure 1:**
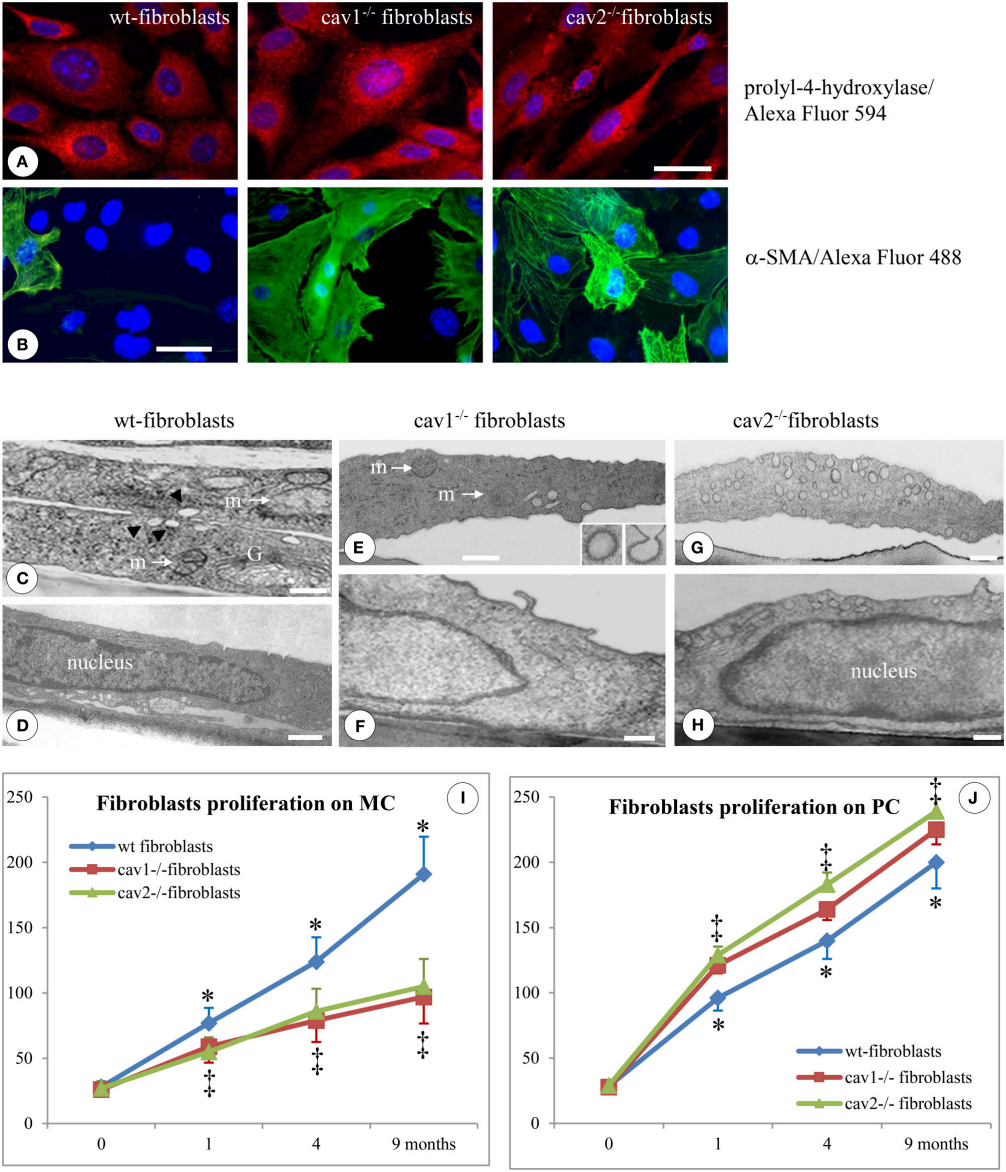
**Specific morphological and proliferative features of isolated mouse lung fibroblasts**. Immunofluorescent staining of fibroblasts isolated from 2 months old mice (1 day after confluence) for the prolyl-4-hydroxilase specific marker reveals its presence in all three types of cells; *n* = 7. **(B)** Staining of isolated fibroblasts (same conditions as in **A**) with α-SMA Ab showing a higher number of positive cells in Cav1^−/−^ and Cav2^−/−^ phenotype; *n* = 8. Bars: 30 μm **(A)**; 25 μm **(B)**. EM morphology of isolated fibroblasts illustrates by sections through the cells outside the nuclear area **(C,E,G)** and sections through the nuclear area **(D,F,H)**. **(C)** Two overlapping wt-fibroblasts display most of regular organelles: Golgi -G-, mitochondria –m-, caveolae (arrows); **(D)** and a nucleus with an continuous rim of condensed chromatin; *n* = 6. **(E,F)** Cav1^−/−^ fibroblasts show total lack of caveolae, while they do have CCVs (insets) all other subcellular organelles, including mitochondria (m), ER spread throughout the cytosol and the nucleus is filled with relaxed chromatin; *n* = 6. **(G)** The Cav2^−/−^ fibroblasts showing a sizable population of caveolae identical in size, number and cellular distribution with wt-fibroblasts, along with a nucleus crammed with relaxed chromatin **(H)**. Bars: 150 nm **(C)**; 200 nm **(D,F)**; 350 nm **(E)**; 300 nm **(G,H)**. **(I)** When isolated fibroblasts were seeded on monomeric collagen-1 (MC) the wt-fibroblasts (blue graph) display a 50% increase in their proliferation vs. Cav1^−/−^ fibroblasts (red graph) and Cav2^−/−^ fibroblasts (green graph). **(J)** Cav-null fibroblasts isolated from the lungs of mice at different ages when plated in polymeric collagen-1 (PC) are more proliferative (>20%) than the corresponding wt-fibroblasts; 0 means 2 weeks of age, while 1, 4, and 9 are months (m) after birth. *n* = 12; ^*^p < 0.05 and ^‡^*p* < 0.01 between different months. In all conditions (MC and PC) the differences between 1, 4, 9 months, and 2 weeks are statistically significant *p* < 0.01. Note also the lack of statistical differences between cav1^−/−^ and cav2 ^−/−^ null fibroblasts in both experimental conditions as well as at different time points.

Lung tissue from Cav1^−/−^, Cav2^−/−^, and wt-mice (C57B strain) was used to isolate fibroblasts that were later characterized morphologically by immunostaining for the expression of prolyl-4-hydroxylase and α-SMA. The anti-prolyl-4-hydroxylase Ab detected the presence of the enzyme in all three types of fibroblasts (Figure 1A). The immunoreactivity for α-SMA varied among the isolated fibroblasts (Figure 1B). Five regions of interest (ROIs) chosen randomly from -three coverslips of each fibroblast type, indicated that the percentage of α-SMA positive cells was 4 ± 0.2%, *p* < 0.05, in wt-fibroblasts; 33 ± 6%, *p* < 0.05 in Cav1^−/−^ fibroblasts and 39 ± 7%, *p* < 0.04 in Cav2^−/−^ fibroblasts. Additional immunostaining studies indicated that the majority of cells, (71% of all the fibroblasts types), were immunoreactive to Thy-1, an antigen specific to fibroblasts subtypes. They also expressed a fibroblast specific antigen recognized by the monoclonal Abs ER-TR7 and stained positive for vimentin, a marker for the mesenchymal origin (not shown). Thus, our immunostaining data strongly suggest that the isolated cells are truly fibroblasts and were used for all experimental procedures.

At the EM level, as expected, Cav1^−/−^ fibroblasts lacked any detectable caveolae (Figures 1E,F), while retaining their clathrin-coated vesicles (Figure 1E, inset). The number and structure of vesicular carriers between wt- and Cav2^−/−^ fibroblasts did not differ (Figure 1C vs. Figure 1G). Morphometric analysis (57 electron micrographs of each type of fibroblasts) of the caveolar numbers per cellular profile revealed 0.5 ± 0.3 caveolae in the Cav1^−/−^ fibroblasts (*p* < 0.001); 17.6 ± 3.4 caveolae in the wt-fibroblasts (*p* < 0.003) and 18.2 ± 4.1 caveolae in Cav2^−/−^ fibroblasts (*p* < 0.002). Using the Neuwman-Keul test we did not found any statistical difference between the number of caveolae in wt vs. Cav2^−/−^ fibroblasts (*p* < 0.27). At this level of resolution, the fibroblasts isolated from the wt- and both Cav-null mice exhibited similar morphological features: (i) a rich endoplasmic reticulum (ER), (ii) a full-bodied Golgi apparatus (G, Figure 1C), (iii) several mitochondria scattered throughout the cytoplasm (m, Figures 1C,E) and (iv) a sizeable smooth nucleus (Figures 1D,F,H). This set of data correlated with the FM staining give us the confidence that the isolated cells are indeed fibroblasts and therefore worthy for the experimental procedures.

The proliferative potential of lung fibroblasts isolated from mice of different ages was examined in both 2D and polymeric collagen-1 matrices in conjunction with the MTT assay, as described in the Section Materials and Methods. Fibroblasts isolated from Cav1^−/−^ and Cav2^−/−^ mice were 50% less proliferative than the fibroblasts isolated from wt-mice when cultivated on 2D matrices (Figure 1I), but they were approximately 20% more proliferative than wt-fibroblasts when cultivated on polymeric collagen matrices (Figure 1J). These results are consistent with previous reports showing that fibroblasts from IPF patients were less proliferative when cultured on 2D matrices (Ramos et al., 2001) or, more proliferative on polymerized collagen as a result of improved integrin signaling (Xia et al., 2008). Thus, our isolated murine lung fibroblasts are endowed with morphological and functional features of the fibroblast phenotype and therefore they are commendable for detection of structural, biochemical and functional changes induced by FasL treatment.

Together, these findings demonstrate that the fibroblasts isolated from Cav-null mice exhibited a characteristic fibroblast phenotype, with proliferative properties, that can be used to identify the molecular determinants involved in the apoptotic-resistance of fibroblasts in IPF. These cells were used throughout the whole work and referred as isolated fibroblasts. First the isolated fibroblasts were used to establish reproducible conditions of apoptosis induction and the main morphological transformations when treated with FasL.

### Cav-null fibroblasts are apoptosis-resistant: morphological evidence

**Figure 2:**
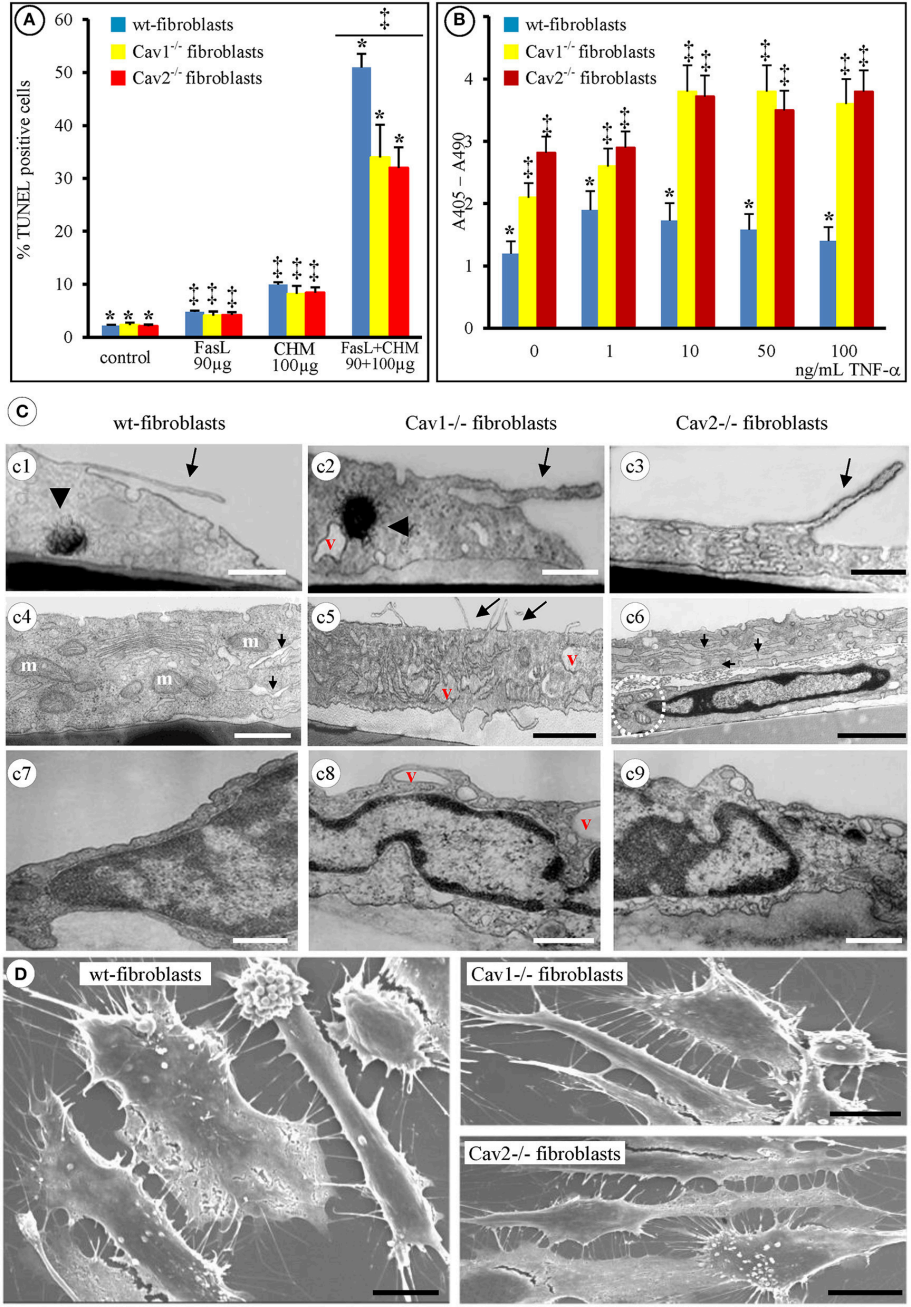
**Apoptotic-resistance characteristics of wt- and Cav-null fibroblasts. (A)** Mouse fibroblasts (wt-, Cav1^−/−^, and Cav2^−/−^) exposed, for 24 h, to either 90 μg/mL FasL or 100 μg/mL CHM are resistant to apoptosis; combined FasL + CHM treatment induces their apoptotic death; *n* = 8, ^*^p < 0.01 vs. control (untreated fibroblasts) and ^‡^*p* < 0.05 vs. FasL or CHM alone. The differences between FasL and CHM treated fibroblast did not reach statistical significance, *p* < 0.24, *n* = 8. **(B)** TNF-α treatment (2 h) induces apoptosis in all fibroblasts in a concentration dependent manner. *n* = 5; ^*^p < 0.001, ^‡^*p* < 0.05. **(C)** By TEM [4 h post FasL **(c1, c2, c3)** and 2 h **(c4, c5, c6)**] all fibroblasts show filopodial extension (**c1, c2, c3, c5**, arrows), display myelinic bodies (**c1, c2**, arrowheads), show increase in the number of mitochondrial units [**c4** (m), **c6** (circled area)], along with enlarged ER (**c4, c6**, arrows) and display increased number of cytoplasmic vacuoles (v; **c2, c5, c8**). Representative electron micrographs demonstrate the nuclear condensation in all three types of fibroblasts **(c7–c9)** and no differences in dismantling of cellular nuclei **(c6, c7, c8, c9)**; Bars: 250 nm **(c1–c9)**; *n* = 6. **(D)** Scanning EM (8 h post-FasL treatment of fibroblasts isolated from 2 months old mice) reveals the extent of cellular blebbing and the abundance of filopodial projections in mouse fibroblasts after FasL-treatment. Bars: 25 μm; *n* = 8.

Preliminary experiments performed with FasL-treated fibroblasts indicated that apoptosis was not induced even with concentrations as high as 2 mg/mL. Apoptosis was not detected when fibroblasts were exposed to 100 μg/mL of the protein synthesis inhibitor CHM alone (Figure 2A). However, apoptotic events were induced in all of the fibroblast types isolated when 100 μg/mL of CHM, followed by 90 μg/mL of FasL were used (Figure 2A). The data in Figure 2A besides establishing the conditions of a reproducible method of FasL-induced apoptosis in isolated mouse lung fibroblasts, also illustrates that the Cav-null fibroblasts were significantly less apoptotic than the wt-fibroblasts. Yet, between Cav1^−/−^ and Cav2^−/−^ fibroblasts no significant differences were determined. In addition, the same CHM + FasL treatment was required to induce apoptosis in isolated lung fibroblasts from mice of different ages (1, 4, and 9 months, not shown). Moreover, the isolated lung fibroblasts, from 1 month old mice, also became apoptotic when treated with different concentrations of TNF-α (1–100 ng/mL). Interestingly, the Cav-null fibroblasts were more responsive, to TNF-α treatment than the wt-fibroblasts (Figure 2B). So, we have established that the isolated fibroblasts respond to FasL only in the presence of CHM and these conditions were utilized throughout our work as Fas-induced apoptosis.

Next, we investigated the detailed morphological changes activated by FasL-induced apoptosis using TEM and SEM. Employing 95% confluent cell cultures and a flat embedding method to prepare the isolated fibroblasts for TEM, we noticed that all three types of fibroblasts underwent cell shrinkage as early as 15 min after FasL treatment that reached its maximum after 1 h (Table 1). During this time interval, the cells began to retract and showed an increased number of filopodia (Figures 2Cc1–c3,c5, long arrows). Morphometric surveys on 150 full cellular profiles, for every type of isolated fibroblast, estimated a total of 349 ± 31 filopodia, (*p* < 0.001) in wt, 351 ± 42, (*p* < 0.002) in Cav1^−/−^ fibroblasts and 351 ± 44, (*p* < 0.001) in Cav2^−/−^ fibroblasts; each of these values are given per μm of cellular perimeter. The following values indicate the number of filopodia per cellular profile: 72 ± 12, (*p* < 0.001) in the wt-fibroblasts, 105 ± 19, (*p* < 0.002) in the Cav1^−/−^ fibroblasts, and 110 ± 26, (*p* < 0.001) in the Cav2^−/−^ fibroblasts. Furthermore, the density of filopodial extensions (the number of filopodia/length of cellular perimeter [#/μm length]) was 0.21 in the wt-fibroblasts, 0.30 in the Cav1^−/−^ fibroblasts and 0.33 in the Cav2^−/−^ fibroblasts indicating that these cellular nanoprotrusions are 1.5-times more numerous in the Cav-null fibroblasts relative to the wt-fibroblasts.

**Table 1:** **The onset of morphological apoptotic alterations of mouse lung fibroblasts induced by FasL + CHM**.

**Morphology**	**Wt-fibroblasts**				**Cav1^−/−^ fibroblasts**				**Cav 2^−/−^ fibroblasts**			
	**1 h**	**2 h**	**6 h**	**24 h**	**1 h**	**2 h**	**6 h**	**24 h**	**1 h**	**2 h**	**6 h**	**24 h**
**CELL SURFACE**												
Shrinkage	+++				+++				+++			
Retraction	+++				+++				+++			
Filopodia	++	+	+	++	+	+	++	+++	±	+	++	+++
Blebbing	±	+	++	+++		+	+	++	±	+	+	++
**CYTOPLASM**												
Myelinoid bodies	+	+	++	±	+	+	+	±	+	++	+	+
Enlarged ER		+	++	++		+	+	+++		+	++	++
**NUCLEUS**												
Condensation		+	++	+++		±	+	++		±	+	+++

*+, <40% of cells; ++, <50% of cells; +++, >60%; ±, inconsistent presence*.

Other evident morphological changes encountered were membrane blebbing, the emergence of myelinic bodies and the development of cytoplasmic vacuoles; all are traits of apoptotic cells. We detected myelinic bodies as early as 30 min after-FasL treatment that apparently, disappeared after 6 h (Figures 2Cc1,c2, arrowheads, Table 1), and a large increase in the number of cytoplasmic vacuoles, after FasL treatment, beginning at 2 h (Figures 2Cc2,c5, Table 1) and lasting more than 24 h (Figure 2Cc8, Table 1). The ER remained intact; however 2 h after FasL treatment became dilated in each of the isolated fibroblast types (Figures 2Cc4–c6, short arrows; Table 1). Assessment of the nuclear morphology revealed that 2 h after FasL exposure, the nuclear chromatin became more condensed (Figures 2Cc6–c9 and Table 1), followed by cell nucleus compaction (size reduction determined by a decrease in circumference and surface area) at later time points. After only 24 h of Fas treatment all of the morphological changes ended in karyorrhexis. Furthermore, SEM analyses of the FasL-treated fibroblasts indicated that between 2 and 6 h post treatment, the fibroblasts shed blebs, which were more numerous in the wt-fibroblasts than in the Cav-null fibroblasts (Figure 2D). Morphometric analysis of the bleb distribution per cellular profiles, performed on 155 wt-fibroblasts, 237 Cav1^−/−^ fibroblasts and 251 Cav2^−/−^ fibroblasts, 48 h post-FasL treatment, indicated 44 ± 17 blebs/μm^2^ of cellular surface, (*p* < 0.002) in the wt-fibroblasts, only 23 ± 9 blebs/μm^2^ of cellular surface, (*p* < 0.004) in the Cav1^−/−^ fibroblasts and 27 ± 6 blebs/μm^2^ of cellular surface, (*p* < 0.001) in the Cav2^−/−^ fibroblasts. Together, these observations suggest that the fibroblasts isolated from Cav-null mouse lungs have an apoptosis-resistant phenotype. Data collected, after our extensive morphological surveys performed on isolated fibroblasts treated with FasL, give us the needed confidence to use these cells for widespread biochemical and molecular analyses in order to unravel the mechanisms and main players behind our morphological findings.

### FasL-induced fibroblasts apoptosis is caspase-dependent

**Figure 3:**
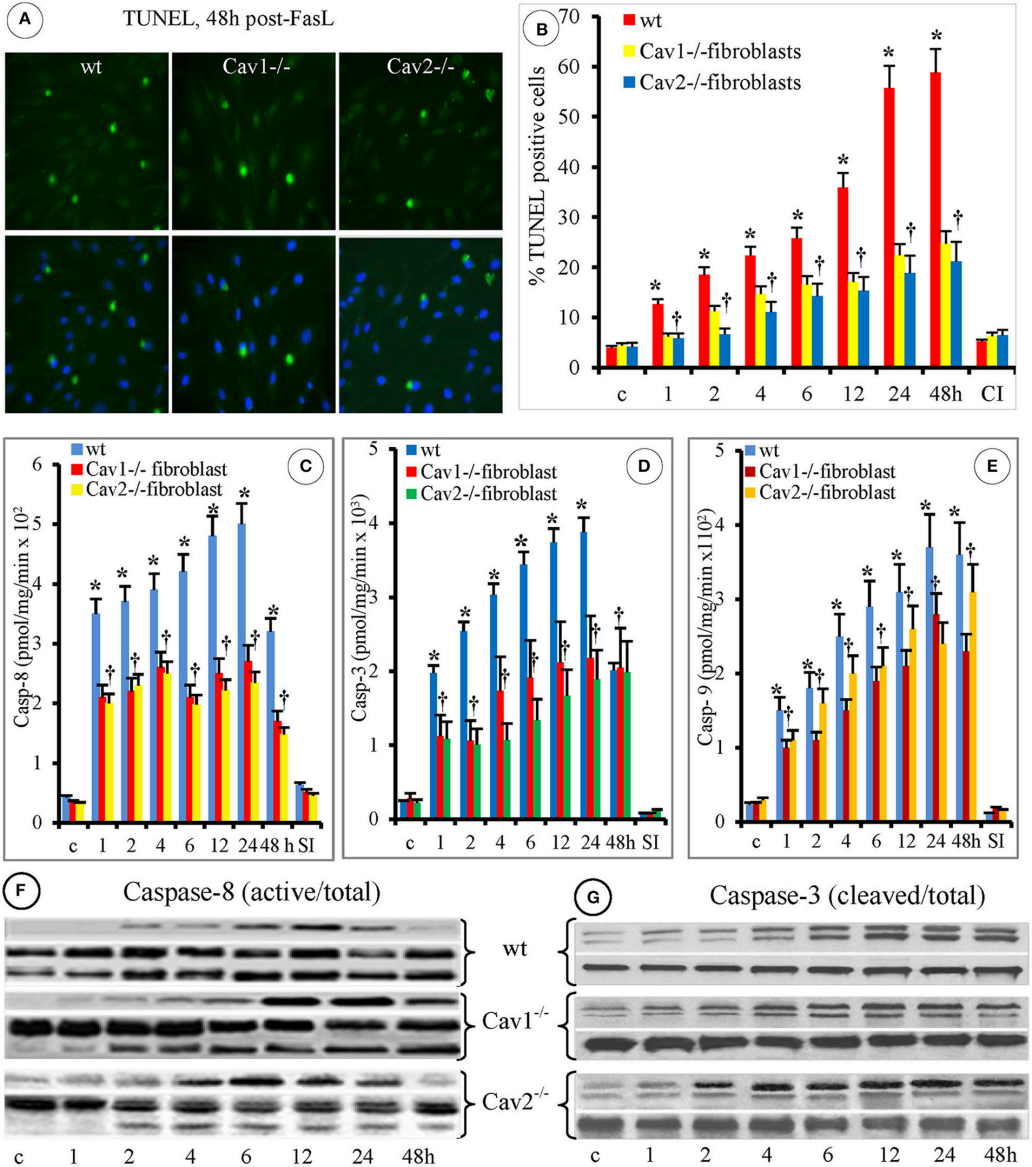
**FasL-induced apoptosis of mouse fibroblasts is caspases-dependent. (A)** The upper panels illustrate the TUNEL staining of mouse fibroblasts at 48 h post- FasL treatment; while the lower panels illustrate the DAPI staining for the detection of cellular nuclei. Bar: 30 mm, *n* = 7. **(B)** Morphometric analyses of TUNEL-positive fibroblasts at different time points after FasL exposure. Note that at 48 h after FasL, the progression of apoptosis is inhibited by 30 μ of pan caspases inhibitor MZ-VAD-FMK (CI); *n* = 4 for every time point and type of fibroblasts, ^*^p < 0.05 when wt-fibroblasts are contrasted with Cav-null fibroblasts. **(C)** Caspase-8 specific activity shows statistical differences between wt- and Cav-null fibroblasts (^*^p < 0.05), and no significant differences between Cav-null fibroblasts, ^†^*p* < 0.26; *n* = 6 for each type of fibroblasts. **(D)** Caspase-3 activity was higher in wt-fibroblasts when compared with cav null fibroblasts, ^*^p < 0.05. Its activity was slightly higher in Cav1^−/−^ compared to Cav2^−/−^ fibroblasts, but without statistical significance, ^†^*p* < 0.14; *n* = 6 for each type of fibroblasts. **(E)** The higher activity of Caspase-9 in the first 24 h reached statistical significance for wt-fibroblasts when compared to Cav1^−/−^ fibroblasts, ^*^p < 0.05, but not when compared with Cav2^−/−^ fibroblasts; *p* < 0.1; its activity was not different between Cav1^−/−^ and Cav2 ^−/−^ fibroblasts, ^†^*p* < 0.22. *n* = 9 for each type of fibroblasts. **(F)** Activation of Caspase-8 (top panels) was evident by 2 h in all types of fibroblasts, peaked between 6 and 12 h post treatments and remained evident in cav null fibroblasts even beyond 24 h, while its total amounts didn't change over time. **(G)** Activation of Caspase 3 (top panels) was delayed, but evident by 4 h and persisted for 48 h. The data from F and G are representative for three experiments for every time point, repeated three times.

Because DNA cleavage into oligonucleotides fragments is a key apoptotic biochemical step, we investigated DNA fragmentation in fibroblasts using the TUNEL assay and microscopic evaluation, 48 h after FasL exposure (Figure 3A). Morphometric analyses performed on 95 ROIs indicated on average, 34 ± 8 TUNEL-positive cells in the wt-fibroblasts, (*p* < 0.003), 21 ± 9 TUNEL-positive cells in the Cav1^−/−^fibroblasts, (*p* < 0.001) and 22 ± 7 TUNEL-positive cells in the Cav2^−/−^fibroblasts, (*p* < 0.002; Figure 3B). In addition, the percentage of apoptotic cells was always lower in the Cav-null fibroblasts when compared to the wt-fibroblasts. The number of TUNEL positive Cav1^−/−^ fibroblasts was 37% lower and the number of TUNEL positive Cav2^−/−^ fibroblasts was 34% lower than that of TUNEL-positive wt-fibroblasts. Interestingly, in the wt- and Cav2^−/−^ fibroblasts, the TUNEL-positive cells were evident 4 h after FasL exposure, while in the Cav1^−/−^ fibroblasts they were apparent after 2 h. An increasing trend of apoptotic cells over time was observed in the wt-fibroblasts. Increases in apoptotic Cav-null fibroblasts, were not prominent, and no statistical significance (*p* < 0.4) was reached between Cav1^−/−^ and Cav-2^−/−^ fibroblasts (Figure 3B). In each of the isolated fibroblast types, the treatment with the specific caspase inhibitor Z-VAD-FMK (30 μM) inhibited DNA fragmentation (Figure 3B). Given this result, we investigated the involvement of caspases in FasL-induced fibroblast apoptosis. First, we investigated the expression of relevant caspases in the isolated fibroblasts, endothelial and epithelial cells by WB of cell lysates. Detection of caspase-2, caspase-3, caspase-6, caspase-7 caspase-8, and caspase-9 in each of the cell types (Figure S1A, Supplementary Data), revealed that the expression of caspase-3 and caspase-8 were always higher in the isolate fibroblasts when compared to the endothelial and epithelial cells (red boxes from Figure S1A, Supplementary Data), and slightly higher in the caveolin null fibroblasts relative to the wt- fibroblasts (Figure S1B, Supplementary Data). As illustrated in panel B from Figure S1 significant differences (*p* < 0.003) were noticed only between isolated fibroblasts and the other two cell types. Next, using the Caspase Colorimetric Protease Assay we discovered that after FasL treatment the caspase-3, caspase-8, and to a lesser extent caspase-9 were activated in all the fibroblast types (Figure S2, Supplementary Data). The FasL-induced activation of caspase-3 and caspase-8 was higher in the Cav-null fibroblasts (*p* < 0.05), while the activation of caspase-9 did not differ significantly between the wt and Cav-null fibroblasts (*p* < 0.33). This most likely reflects the different levels of caspase expression illustrated in Figure S1A, Supplementary Data. To resolve this discrepancy we determined the specific activity of the three activated caspases (3, 8, and 9).

Assessment of the specific enzymatic activity of the different caspases after-FasL treatment, revealed that caspase-8 (Figure 3C) was activated first in all of the isolated fibroblasts, persisted for 24 h and its activity was less than half at 48 h. Preliminary experiments (not shown) indicated that high caspase-8 activity could be detected in the first 10 sec, reaches near maximal values at 10 min after FasL stimulation and then its activation profile is shown in Figure 3C. Caspase-3 was activated usually in the wt-fibroblasts (Figure 3D) more than in Cav-null fibroblasts, lasted 24 h, and retained more than 50% of activity at 48 h. Caspase-9 activation was lower (Figure 3E), peaked at 24 h but remained active more than 48 h when compared to caspase-8 and caspase-3. It became obvious that: (i) the activation of caspase-8 is rapid and sustained, up to 48 h after stimulation, (ii) the activation of caspase-3 is more robust (>1.5 orders of magnitude greater than the other two caspases), but not as prolonged (up to 24 h), (iii) the activation of caspase-8 and caspase-9 was observed mainly in the Cav-null fibroblasts and (iv) the activity of all three caspases was inhibited by the specific peptides [(SP), Figures 3C,D,E]. Because caspase-8 is the initiating protease and caspase-3 is the executing protease, we investigated their activation using WB. The appearance of the cleavage fragments of caspase-8 (Figure 3F) and caspase-3 (Figure 3G) in the cell lysates of isolated fibroblasts, followed the same activation time course detected by measuring their specific activity. The presence of fast activated caspase-8 in isolated fibroblast usually reflects an activation of the mitochondrial pathway and therefore we investigated the status of the mitochondrial continuum from isolated fibroblasts.

### Fibroblasts' mitochondria participate in FasL-induced apoptosis

**Figure 4:**
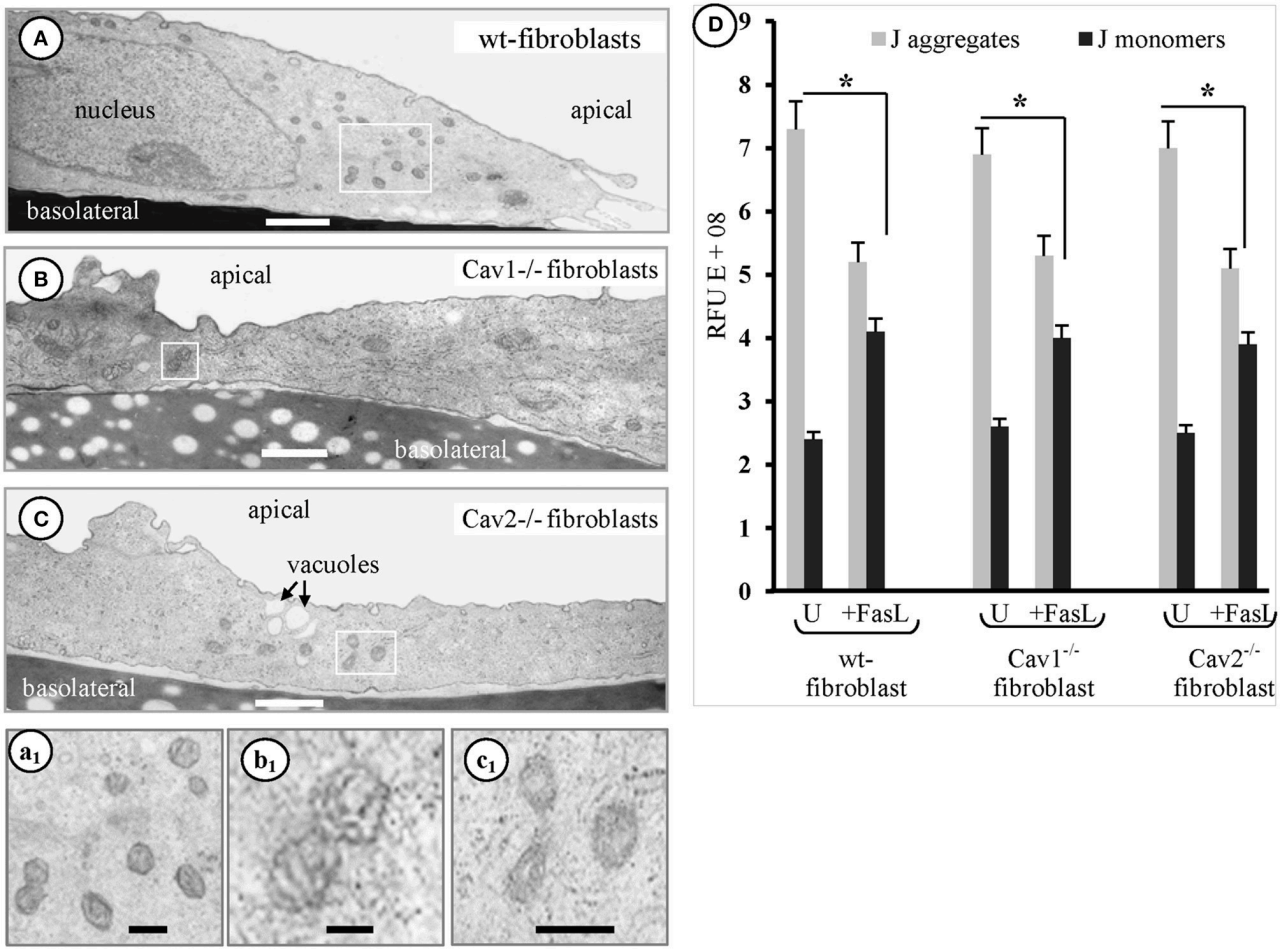
**Fibroblasts mitochondria participate in FasL-induced apoptosis**. Representative electron micrographs of the mouse wt-fibroblasts **(A, a1)**, Cav1^−/−^ fibroblasts **(B, b1)** and Cav2^−/−^ fibroblasts **(C, c1)** demonstrate the increased number of small mitochondrial units scattered all over the cytoplasm after FasL treatment; Bars: 350 nm **(A–C)**; 500 nm **(a1,b1,c1)**; *n* = 6 for each type of fibroblasts. **(D)** Adherent fibroblasts, treated with FasL and CHM (24 h) and then incubated with JC-1 staining solution shows that untreated (U) cells have lots of J-aggregates, and that there are less J-aggregates and more monomeric JC-1 after FasL treatment, indicative of an altered ΔΨm. Statistical significant differences (^*^p < 0.05) were found between FasL-treated and untreated fibroblasts, but when differences between groups were determined they did not reach statistical significance (*p* < 0.47); *n* = 6 for each condition and each type of fibroblasts.

Another organelle affected by FasL exposure in the isolated fibroblasts, was the mitochondrion. EM analyses of the fibroblasts, 1 h after FasL exposure, frequently revealed elongated and condensed mitochondrial profiles along with increased cellular density (Figure 4). In fact, the EM surveys indicated that the earliest detectable morphological event that occurred after-FasL exposure was the appearance of a fragmented mitochondrial continuum followed by cells shrinkage. These two events began after 15 min and became well-defined by 30 min. To better define the mitochondrial alterations, we then measured its membrane potential (ΔΨm) using the JC-1 assay kit and the ratio of the fluorescence intensity. J-aggregates/monomers were used to calculate changes in ΔΨm as induced by apoptotic treatment. We found that ΔΨm was lost within the first hour after FasL exposure (Figure 4D). Thus, all studies were performed within 1 h of FasL exposure. Since the EM morphological surveys, of the cells exposed to FasL, suggested an increased number of mitochondrial profiles per cell, we determined whether the number of mitochondrial DNA copies /cell changed after the induction of apoptosis. The presence of the mitochondrial transcription factor (mtTFA), a key regulator of mtDNA replication, with a level that is proportional to mtDNA (Choi et al., 2001), was investigated using qPCR with 4 ng of each total cellular DNA and linearized pGEMTE-12S vector. A logarithmic regression of the standard sample was used to determine the number of mtDNA copies per pg of total cellular DNA (Table 2). The average number of mtDNA copies per cell (Table 2) was 863 in the wt-fibroblasts, 889 in the Cav1^−/−^ fibroblasts and the 883 in the Cav2^−/−^ fibroblasts. The FasL-treated and untreated fibroblasts did not differ significantly (*p* = 0.4 in wt-, 0.23 in Cav1^−/−^ lines 32 and 0.37 in Cav2^−/−^ fibroblasts). There were no differences between different types of fibroblasts. Our findings indicate that the total amount of mtDNA/cell and/or the mtDNA/total cellular DNA ratio do not change after FasL-induced apoptosis in any type of the fibroblast types (Table 2). Therefore, the status of the mitochondrial continuum and not the number of mitochondria was affected. Similar increases were observed in the apparent density of mitochondrial units along with the loss of its membrane potential during the first hour after FasL in fibroblasts isolated from mice of different ages (not shown). Hence, we concluded that after FasL treatment, the mitochondrial mass/cell does not change and there is more isolated mitochondrial units/cell, due to the widespread fragmentation of the mitochondrial continuum. Fast activation of the initiator protease- caspase-8- (s to 10 min), followed by the fragmentation of mitochondrial continuum starting 10–15 min after FasL stimulation, is a certain sign that the mitochondrial pathway take part in isolated fibroblasts response to CD95 apoptosis signaling.

**Table 2:** **Mitochondrial DNA in cultured murine fibroblasts**.

**Cells**	**wtF**		**Cav1^−/−^ fibroblasts**		**Cav2^−/−^ fibroblasts**	
	**−FasL**	**+FasL**	**−FasL**	**+FasL**	**−FasL**	**+FasL**
Total DNA mass^*^ (pg/cell)	8.1 ± 1.8 (*n* = 6)	9.4 ± 0.87 (*n* = 5)	9.4 ± 1.1 (*n* = 5)	10.4 ± 0.8 (*n* = 7)	9.83 ± 1.1 (*n* = 6)	10.5 ± 0.9 (*n* = 7)
mtDNA- copies (per cell)	844 ± 192 (*n* = 6)	882 ± 234 (*n* = 5)	863 ± 191 (*n* = 5)	915 ± 188 (*n* = 7)	857 ± 205 (*n* = 6)	908 ± 155 (*n* = 7)

* *The predicted mass of a diploid mouse genome of 2.8 × 10^9^ base pairs is 3.3 pg/cell*.

### Phosphatidylserine, M30 and CD95 alterations were similar in the Cav-null and wt-fibroblasts but with distinct temporal manifestations

The morphological changes occurring on the cell surface of FasL-treated fibroblasts were investigated by FM for the detection of Annexin V, M30, and CD95.

**Figure 5:**
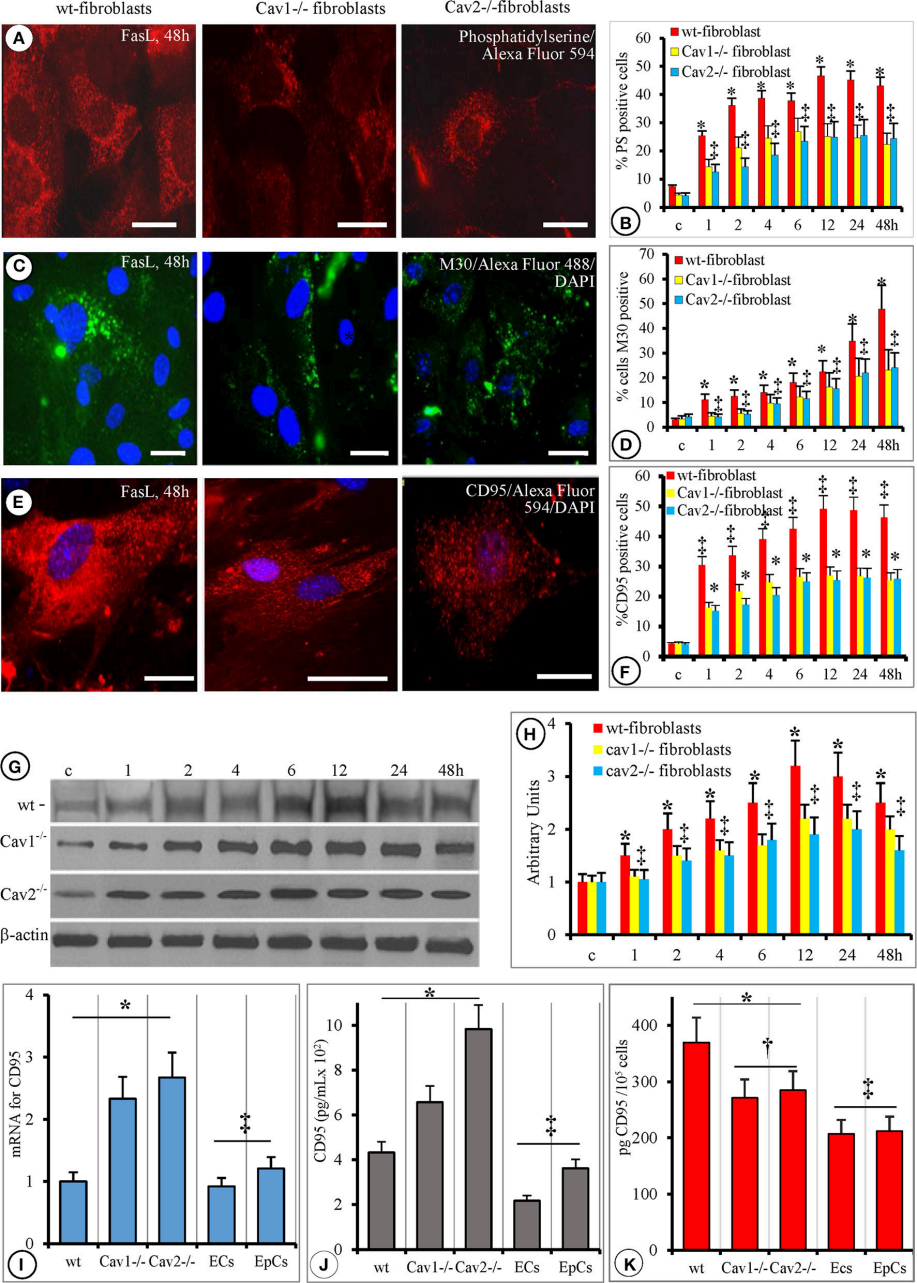
**Phosphatidylserine, M30 and CD95 alterations are similar in cav-null and wt-fibroblasts but with a distinct temporal manifestation. (A)** Representative Annexin V staining of mouse fibroblasts (wt-, Cav1^−/−^, Cav2^−/−^, as indicated) demonstrates the presence of PS on the external leaflet of the plasmalemma proper after FasL stimulation; the images are representative for mice fibroblasts isolated at 4 months old. Note the decreased fluorescence displayed by Cav-null fibroblasts. Bars: 25 μm, *n* = 7. **(B)** Quantitative assessment of PS-positive cells on 70 ROIs, collected from six different experiments for every type of fibroblasts; ^*^p < 0.05 Cav-null vs. wt-fibroblasts, while no statistical differences (^‡^*p* < 0.22) were found between Cav1^−/−^ and Cav2^−/−^ fibroblasts; **(C)** demonstrative images demonstrating the presence of M30 antigen, at 48 h, after FasL treatment in fibroblasts isolated from 4 months old mice. Bars = 20 μm, *n* = 6. **(D)** Quantification of M30-positive cells on 45 ROI for every type of fibroblast. Analysis of M30 presence did not reach statistical significance between Cav1^−/−^ and Cav2^−/−^ fibroblasts; ^‡^*p* < 0.31, but it was significant when wt-fibroblasts are compared to control (untreated) cells, ^*^p < 0.05; *n* = 6 for every time point and cell type. **(E)** CD95/FasL presence on fibroblasts isolated from 4 months old mice revealed the same pattern of its surface distribution (scattered immunoreactive puncta) on all types of fibroblasts. Bars: 25 μm, *n* = 8. **(F)** Semi-quantitative evaluation (percentage of CD95/FasL immunoreactive cells out of the total number of cells) performed on 55 ROI, for each type of fibroblasts, over time reveals that the only significant difference was between wt- and cav null fibroblasts (^*^p < 0.05) and among the untreated (control) and FasL-treated fibroblasts. **(G)** WB analysis of Fas bound to the membrane fraction shows fluctuation on the amounts over a 48 h FasL treatment; *n* = 4 for every type of fibroblast and time point and the experiments were repeated three times. The graph on **(H)** shows the quantitative aspect of membrane bound Fas, and points to the fact that there is no statistical significance between wt and cav-null fibroblasts, *p* < 0.25 as well as between Cav1^−/−^ and Cav 2^−/−^ fibroblasts, *p* < 0.3. **(I)** qPCR evaluation of mRNA for CD95/FasL showing that Cav-null fibroblasts synthesize more CD95 than wt-, ECs, and EpCs, *n* = 6, *p* < 0.05, while no statistical significance was detected between ECs and EpCs, *p* < 0.25. **(J)** ELISA quantification of soluble form of CD95/FasL (released into culture media) indicating that the fibroblasts secrete more CD95 than the lung ECs and EpCs. *n* = 8, ^*^p < 0.01 when fibroblasts are compared to ECs and EpCs and ^‡^*p* < 0.05 between different types of fibroblasts. **(K)** Cell surface expression of CD95 as detected by cell ELISA demonstrated its presence at a higher levels in all fibroblasts than in the ECs and the EpCs, ^*^p < 0.05; the fact that are no significant differences in FasR amounts between ECs and EpCs, ^‡^*p* < 0.27 and the finding that the surface CD95 was less in Cav-null fibroblasts than in the wt-fibroblasts, ^‡^*p* < 0.02.

Annexin V staining, of FasL-treated fibroblasts, used to detect the presence of PS revealed full bodied expression after1 h that became evident at 2 h, reaching its maximum at 6 h. Its expression remained elevated for 48 h (Figures 5A,B). Interestingly, the expression of PS 3–6 h after FasL treatment coincided with cell membrane blebbing (Table 1). This finding was in agreement with the weakening of interactions between the cell membrane and the cytoskeleton. The staining was weaker in the wt-fibroblasts compared to Cav-null fibroblasts after FasL treatment (Figure 5A). Morphometric analyses indicated that the number of PS-positive cells in the Cav-null fibroblasts was always 50% lower than in the wt-fibroblasts (Figure 5B). These findings: a weaker staining in the wt-fibroblasts compared to Cav-null fibroblasts, but a lower number of PS positive cells in the Cav-null fibroblasts, after FasL, were indicative of a constitutive weakening of membrane-cytoskeleton interactions in the Cav-null phenotype, that seems to be ones of the salient differences between the wt and Cav-null fibroblasts.

The initial cellular shrinkage and retraction were accompanied by changes in the cytoskeletal elements as indicated by M30 Ab staining (Figure 5C) that specifically recognizes the newly formed caspase-cleaved epitope of cytokeratin-18. The emergence of the M30 epitope was well-established 1 h after FasL treatment in all three fibroblast types. The quantification of M30-positive cells after FasL treatment demonstrated that the number of M30-positive cells in the Cav-null fibroblasts there was 40% lower, on average, than in the wt-fibroblasts (Figure 5D).

Because the expression of Fas/CD95 may play a critical role in FasL-induced apoptosis, we investigated its cellular distribution by FM (Figures 5E,F) and WB (Figures 5G,H). For the presence of sFasL we used PCR and ELISA (Figure 5J). Post-FasL treatment, FM indicated a strong signal for Fas/CD95 on the surface of wt-fibroblasts and a much less intense signal on the surface of Cav-null fibroblasts (Figure 5E). However, the number of Fas/CD95-positive cells was 35% lower in the Cav-null phenotype than in wt-fibroblasts (Figure 5F) at different time points. WB analysis of sCD95 distribution indicated similar results: (i) increases in membrane-bound sCD95 (Figure 5G), (ii) 30–35% lower amounts of CD95 in Cav-null fibroblasts (Figure 5H), (iii) comparable dynamic development of CD95 expression over time revealed by FM (Figures 5G,H), and (iv) a lower content (pg CD95/cell) of the receptor in Cav-null fibroblasts (Figure 5K). Unexpectedly, when the mRNA levels of Fas/CD95 were assessed with PCR (Figure 5I), we found that its levels were 1.5 times greater in the Cav-null fibroblasts relative to the wt-fibroblasts, lung ECs and EpCs. Because the increased levels of CD95/Fas mRNA do not translate into increased surface expression, we determined the concentration of sFas in the cell growth media using ELISA. We found an approximately 2.3-fold increase of sFas in the media from Cav2^−/−^ fibroblasts and only a 1.4-fold increase in the media from Cav1^−/−^ fibroblasts. The ECs and EpCs secreted the least amount of sFasL and displayed lower levels of surface CD95 when compared to all types of isolated murine lung fibroblasts (Figures 5H,K). For CD95, a “FADD-only” recruiting receptor that generates apoptosis signals or triggers non-apoptotic signaling events, the apoptosis induced signals are demonstrated to be produced only when the receptor molecules are located in membrane rafts. In preliminary experiments we did not found, by immunostaining of isolated fibroblasts, co-localization of CD95 with cav1, cav2, and clathrin. So, we concluded that in murine lung fibroblasts CD95 it is localized within membrane rafts and our data are in agreement with published data in other cell types.

As the quantities of sFasL do not always correlate with increased apoptosis resistance and since the isolated Cav-null fibroblasts display the lowest cell surface amounts of CD95 we consider that the FasR receptor in the fibroblasts is not the main contributor to the apoptosis resistance to FasL treatment; it function as a trigger whose signaling effects are fine-tuned by other contributing molecules. So, we investigated the contribution of other molecules: IAPs, Bcl-2 family members, DDR1, and cFLICE in order to reveal the necessary molecular machinery responsible of the recognized fibroblasts apoptosis resistance.

### XIAP is a key component of fibroblast' resistance to fas-induced apoptosis

**Figure 6:**
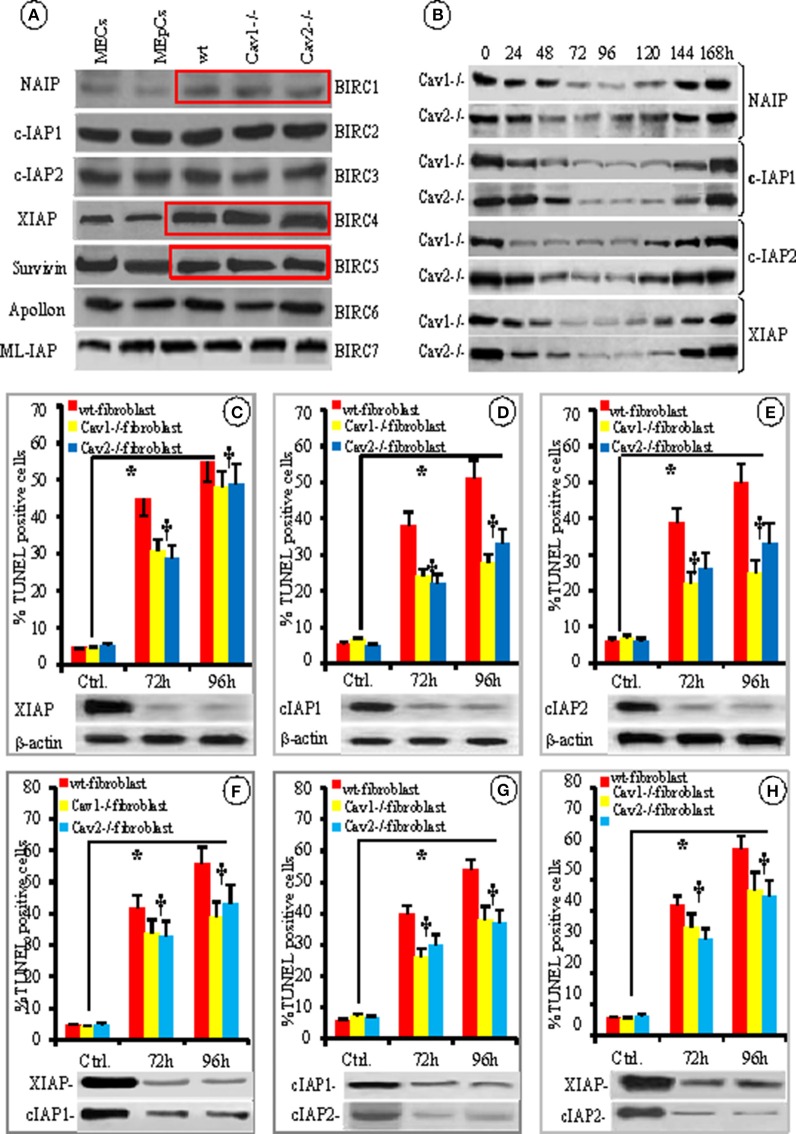
**IAPs involvement in fibroblast resistance to apoptosis. (A)** Representative WB using specific Abs for different IAPs protein expression in the mouse lung cells–ECs, EpCs, wt-, Cav1^−/−^ and Cav2^−/−^ fibroblasts, as indicated; *n* = 6 for each IAP. **(B)** The time course and transient nature of siRNA-mediated knockdown of four BIRCs: NAIP, cIAP1, cIAP-2, and XIAP- in Cav-null fibroblasts; *n* = 4 for each IAP and Cav-null fibroblasts. The effects of individual knockdown as indicated–XIAP **(C)**, cIAP1 **(D)** and cIAP2 **(E)**, *n* = 4 experiments for every type of fibroblasts and every time point, ^*^p < 0.05 between controls and cav –null fibroblasts but ^†^*p* < 0.1 in between Cav1^−/−^ and Cav2^−/−^ fibroblasts. The combined knockdown–XIAP/cIAP1 **(F)**, cIAP1/cIAP2 **(G)**, and XIAP/cIAP1 **(H)** effects in fibroblasts resistance to FasL-induced apoptosis; *n* = 6, ^*^p < 0.05 when compared to untreated fibroblasts.

Because the members of the IAP and Bcl-2 families of apoptotic-related proteins regulate mitochondrial triggered apoptosis, we investigated their expression in the cellular lysates of isolated fibroblasts by comparing them to murine ECs and EpCs using WB (Figure 6A). The presence of IAPs “the masters of cellular apoptosis” was examined. We found that the expression of NAIP/BIRC1 and c-IAP1/BIRC2 was slightly increased in fibroblasts (12 and 10%, respectively); while the expression of IAP2/BIRC3 was 11% lower than in resting Cav-null fibroblasts (Figure 6A, red boxes) when compared to ECs and EpCs. The expression of survivin/BIRC5 was dynamic. At times, the expression was 15% lower and at other times was more than 20% higher in fibroblasts. These fluctuations, most likely depend on: (i) the phase of the cell cycle at the time of cell collection and lysates preparation, as reported previously (Otaki et al., 2000), (ii) gene transcriptional status as shown by its upregulation by NFkB (Mita et al., 2008) or (iii) gene upregulation by the ILGF1/mTor pathway (Vaira et al., 2007), by c-H-Ras (Sommer et al., 2003), and Wnt-2 (You et al., 2004). Noticeably, XIAP/BIRC4 was the only IAP that exhibited striking difference in expression; it was expressed 2–3 times more in wt-fibroblasts than in ECs. Furthermore, the expression of XIAP/BIRC4 was 1.5–2 greater in the Cav-null fibroblasts than in wt-fibroblasts (Figure 6A, Figure S3A from Supplementary Data).

Next, we used specific siRNAs to knockdown the first four BIRCs to evaluate their effects on fibroblast apoptosis. Efficient knockdown was achieved 72–96 h post-siRNA transfection. Protein expression was recovered in all cases by 144 h post-siRNA transfection (Figure 6B). Because the IAPs BIRC-2, 3, and 4 are thought to control apoptosis (Vaux and Silke, 2003; Moodley et al., 2004; Larsson et al., 2008; de Almagro and Vucic, 2012) we knocked down each of them individually or in pairs and assessed their effect on fibroblast apoptosis using TUNEL 72 and 96 h later. The knockdown of individual IAPs did affect the proportion of apoptotic wt-fibroblasts, but it was statistically significant only for XIAP. XIAP knockdown made the wt-mouse fibroblasts more sensitive (>35%) to FasL-induced apoptosis (Figure 6C). The knockdown of BIRC2 (*p* < 0.38, Figure 6D) or BIRC3 (*p* < 0.22, Figure 6E) did not significantly impact fibroblast apoptosis. To better define the involvement of IAPs in fibroblast apoptosis, double knockdowns were performed (Figures 6F,G,H). The TUNEL assay did not indicate any additive effects on fibroblast apoptosis. In the case of XIAP-IAP1 (Figure 6F) and XIAP-IAP2 double knockdowns (Figure 6H), the apoptosis-resistance was similar to XIAP knockdown alone, (35% decrease in fibroblast apoptosis-resistance), while the cIP1-cIAP2 double knockdown did not exhibit any differences. Thus, our results demonstrate that the fibroblasts constitutively express more XIAP, and the involvement of this increased expression in establishing fibroblast resistance to FasL-induced apoptosis was more than 35%. It was surprising that XIAP knockdown was involved only 35% in the apoptosis-resistance to FasL treatment and we decided to investigate other molecules reported to participate in fibroblast apoptosis-resistance.

**Figure 7:**
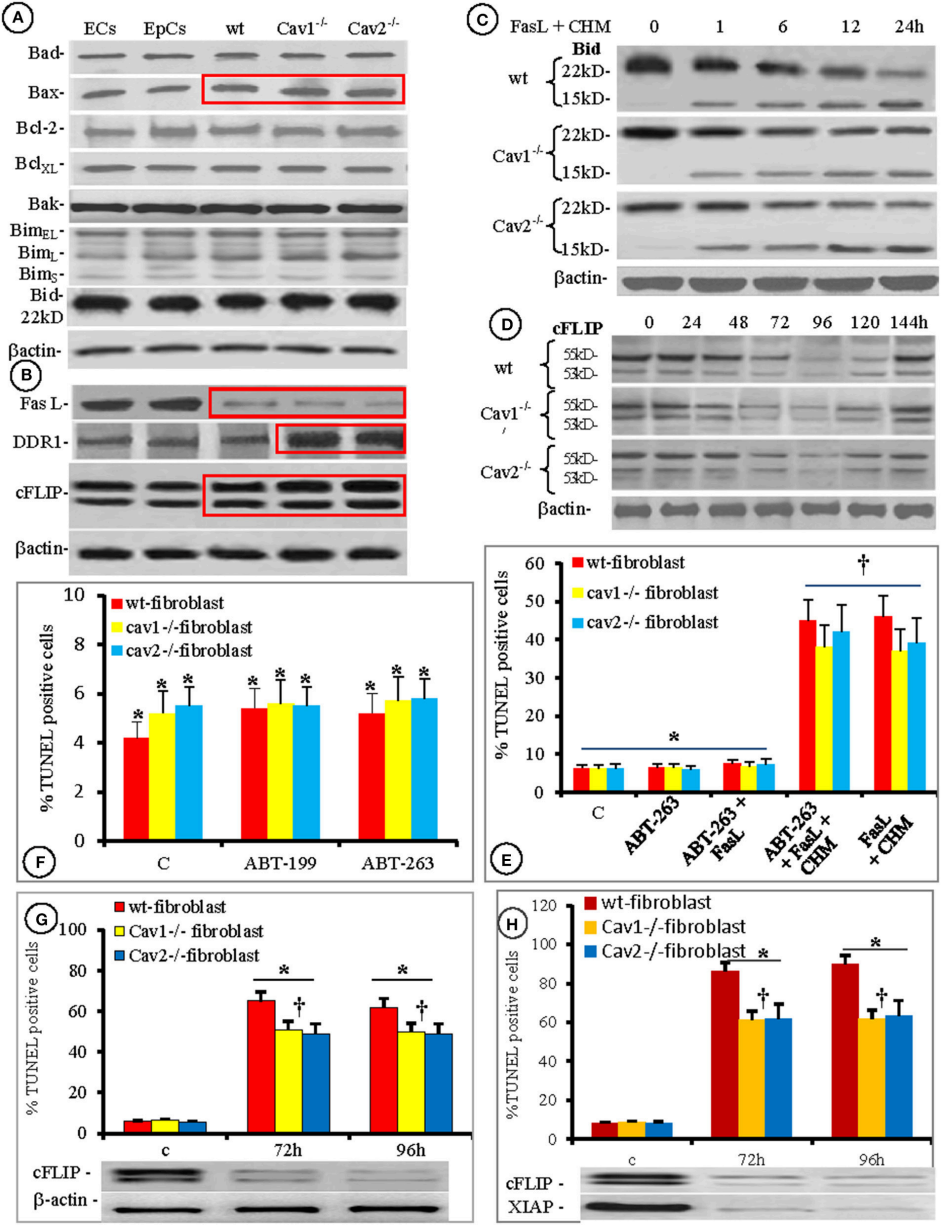
**Contribution of Bcl-2 family members, cFLIP, and XIAP to fibroblasts' increased resistance to apoptosis**. Protein expression of seven members of Bcl-2 family of apoptotic proteins shows that in different types of lung cellular populations, only Bax is constitutively overexpressed (red box); *n* = 5 for each type of cells. **(B)** Expression of other apoptotic proteins, in the same types of isolated cells showing (red rectangles): FasL downregulation, DDR1 upregulation–especially in Cav-null fibroblasts, and cFLIP upregulation in all fibroblasts; *n* = 5 for each type of cells. **(C)** Presence of cleaved form of Bid (15 kD fragment on isolated fibroblasts treated with FasL and CHM), is easily detected with a specific anti tBid Ab; *n* = 7 for each time point. **(D)** The transitory effect of siRNA silencing of cFLIP showing its temporary down regulation between 72 and 120 h; *n* = 5 for each type of fibroblasts and every time point. **(E)** Pharmacologic inhibition of Bcl-2 **(E,F)** shows no effect on fibroblasts resistance to apoptosis by the broad specific ABT-236 **(F)** or by the more specific ABT-199 **(E)** as detected by the TUNEL reaction; *n* = 8 for every type of fibroblast, ^*^p < 0.3. However, ^†^*p* < 0.001 when the fibroblasts were treated with FasL + CHM even in the presence of ABT-236; *n* = 6 for every type of fibroblasts and the experiments were repeated three times. **(G)** Increased apoptotic susceptibility of cells to FasL in cFLIP knockdown fibroblasts; *n* = 7, ^*^p < 0.01 when compared with the controls and ^†^*p* < 0.05 in between wt and cav-null fibroblasts. **(H)** Double XIAP/ cFLIP knockdown shows an additive effect on apoptosis resistance to FasL treatment. *n* = 7, ^*^p < 0.05 when compared to control and ^†^*p* < 0.01 in between wt and Cav-null fibroblasts.

Thus, we examined the expression of other apoptotic proteins (Bad, Bax, Bcl-2, Bcl_XL_, Bak, Bim_EL_, Bim_L_, Bim_s_, and Bid). Among them, only Bax expression was <20% higher in all isolated fibroblasts, when compared to the ECs and EpCs (Figure 7A, boxed area). Because the overexpression and/or function of Bcl-2 were connected to apoptosis resistance in rat heart fibroblasts (Mayorga et al., 2004), we investigated its functionality during FasL triggered apoptosis of mouse lung fibroblasts using pharmacological inhibition. As shown in Figures 7E,F, the inhibition of Bcl-2 by ABT-236 (a broad range inhibitor) did not impact FasL-induced apoptosis. Moreover, the percentage of induced apoptosis did not differ from the conditions used throughout the paper (last two columns of Figure 7F). Pretreatment of isolated mouse lung fibroblasts with the more specific inhibitor ABT-199 also did not affect FasL-induced apoptosis (Figure 7E). Thus, our results prove that Bcl-2 is not overexpressed and its functional inhibition does not modify the apoptotic resistance of isolated mouse lung fibroblasts.

One of the most exploited Fas-triggered pathway leading to mitochondrial membrane permeabilization, cytochrome c release, and apoptosis, involves caspase-8 activation, Bid cleavage and the translocation of a 15 kDa tBid fragment to the mitochondrial membrane to facilitate Bax activation (Kantari and Walczak, 2011). Therefore, we investigated the status of Bid in the isolated fibroblasts after FasL-treatment. While the expression of Bid was the same in all lung cell types investigated (Figure 7A) after FasL treatment, we consistently detected the 15 kDa fragment Bid cleavage product, after 1 h. We were able to detect this fragment beyond 24 h (Figure 7C).

It has been suggested that fibroblast resistance to apoptosis is mediated by DDR1 (Matsuyama et al., 2007), FasL (Kuwano et al., 2002; Moodley et al., 2004; Larsson et al., 2008; Kopinski et al., 2011) and c-FLIP (Safa, 2012), therefore we also explored their presence in the isolated fibroblasts and compared their levels to those measured in ECs and EpCs. We found that the expression of all three proteins was constitutively modified (Figure 7B). FasL, bound to the cell membrane was downregulated in each of the fibroblasts types (red rectangle in upper panel from Figure 7B). DDR1 expression in the wt-fibroblasts did not differ from the ECs and EpCs, while a 2- to 3-fold increase in expression was observed in the Cav-null fibroblasts (red boxed area in second panel). c-FLIP expression was augmented in all fibroblasts (1.5-fold increase in wt-fibroblasts vs. the EpCs and ECs and >2-fold increase in Cav-null fibroblasts vs. wt-fibroblasts, red bounded area in the third panel of Figure 7B and Figure S3B from Supplementary Data.

As in the case of individual IAPs we knockdown the DDR1 using specific siRNA, and found that its influence in fibroblast apoptosis resistance, detected by the TUNEL assay, was less < 10% and remained in the same range (around 10%) even when double knockdown with BIRC1 and BIRC2 and BIRC5/XIAP were tried (not shown). Thus, we silenced c-FLIP and the results follow.

Knockdown of c-FLIP expression was transitory. Maximal downregulation occurred after 96 h (>85%) in all of the fibroblast types and full recovery was achieved after 144 h (Figure 7). The sensitivity of fibroblasts to apoptosis increased by >35% after c-FLIP knockdown (Figure 7D), while double XIAP/c-FLIP knockdown increased the sensitivity to FasL treatment in fibroblast by almost 80% (Figure 7E). Based on these data we concluded that the apoptosis resistance of lung fibroblasts to FasL-induced apoptosis was more than 80% dependent on XIAP and c-FLIP expression, while the presence of other molecular signatures such as DDR1, p53, sFas and the activation/deactivation status of other survival pathways explain the remaining 20%.

## Discussion

Apoptosis is the best characterized process of programmed cell death that results in the demise of the cell without the activation of innate immune responses (Danial and Korsmeyer, 2004). Since cell death was first described in the 1960s, a number of different death mechanisms have been described and classified, based on both morphological and biochemical criteria. Apoptosis, a controlled cessation of cellular processes, is characterized at the cellular level by cell shrinkage, nuclear condensation/fragmentation, and membrane vesiculation/blebbing (Wolfs et al., 2005). The activation of the Fas receptor induces apoptosis *in vivo* in cell types that include hepatocytes, lymphoid, endothelial, and glomerular and lung epithelial cells (Matute-Bello et al., 2001; Janin et al., 2002).

Our study determined that lung mouse fibroblasts are resistant to FasL ligation unless CHM is added, as previously reported in human fibroblasts (Tepper et al., 2000; Santiago et al., 2004). We also show that lung mouse fibroblasts respond differently to another apoptosis inducer –TNF-α. Even if TNF-receptor1 (TNFR1) and FasL use homologs signaling pathways, we suggest that the dissimilarity arises because the two receptors utilize different proximal signaling molecules to activate the caspase pathway. TNFR1 requires the adapters: Tumor necrosis factor receptor type 1-associated DEATH domain protein (TRADD) and receptor interacting protein (RIP) to transduce signals to the Fas-Associated protein with Death Domain (FADD) and the downstream caspase 8. The fine tuning of the responses mediated by the TNF-receptor 1 are regulated by its internalization as long as its compartmentalization dictates which signaling pathways used by the receptor are utilized. The pro-apoptotic signaling emanates primarily from intracellular vesicles and requires receptor internalization, whereas the non-apoptotic signaling could start at the plasma membrane and it was shown that only late steps in receptor signaling are internalization dependent. Our data suggest that the differences in response of isolated fibroblasts to FasL and TNF could be explained by the way in which the two receptors signal.

Activation of Fas receptor (FasR), triggers apoptotic, and non-apoptotic signaling pathways that diverge at the plasmalemma level. The FasR, a “FADD-only” recruiting molecule does not require TRADD or RIP for signaling. Instead, FasR can interact directly with FADD, leading to the activation of caspase 8 inside of the newly formed DISC, as our data suggest that happens in the murine lung fibroblasts. FasL binds to the CD95, localized in membrane rafts, where starts to form stable FasR micro-aggregates which coalesce into larger signaling platforms. At this level the apoptosis and no-apoptosis signaling pathways are separated. The apoptosis signaling complexes need to be internalized into the endosomal compartment where high levels of active DISC are found. Inside the DISC caspase-8 dimerize and this is the first step in the activation of this protease, then the active molecule is converted in its mature heterotetrameric form by auto-proteolytic maturation and finally is released from the DISC. Active caspase-8 initiates the apoptotic cascade. Thus, the two receptors are using same basic signaling molecule but in a very different combination which is fine tuned for specificity, intensity and length of response by additional molecules. These fine tuners are the molecules making the difference in the response of the two receptors.

Importantly, the time needed for wt-fibroblasts or Cav-null fibroblasts to undergo FasL-mediated apoptosis was 6–7 h, a result that is in agreement with previously published work on different cell types (Duke et al., 1986) and human fibroblasts. In this context, our data demonstrate that the resistance to apoptosis of Cav-null fibroblasts depends not only on the expression of anti-apoptotic proteins, but also on the increased production of sFas which still plays a small role in the anti-apoptotic process. These findings are similar to those reported in human fibroblasts isolated from IPF patients (Moodley et al., 2004; Buhling et al., 2005). Studies of human IPF have established that decreased apoptosis of modified fibroblasts/myofibroblasts plays a role in the persistence of fibrotic lesions leading to organ dysfunction (Barbas-Filho et al., 2001; Buhling et al., 2005; Plataki et al., 2005). Our data, spanning over 9 months, also indicate that the Cav-null fibroblasts/myofibroblasts are present at all times in murine lungs that display a fibrotic phenotype.

Both morphological changes (Moodley et al., 2004; Thannickal and Horowitz, 2006) and common biochemical markers (Tanaka et al., 2002; Moodley et al., 2004; Wynes et al., 2011; Ajayi et al., 2013) were examined during human IPF fibroblast apoptosis; however, few studies have examined apoptosis in mouse lung fibroblasts (Moodley et al., 2004) as this field is still an active area of investigation. We report that the number of wt-fibroblasts undergoing FasL-induced apoptosis was significantly higher than in Cav-null fibroblasts. These findings indicate an increased resistance to apoptosis in Cav-null fibroblasts. In addition, isolated Cav-null fibroblasts had a greater proportion of α-SMA-positive cells than the wt-fibroblasts, suggesting an increase in myofibroblasts-like cells. However, the presence of a constitutively increased number of myofibroblasts may reflect a more rigid environment rather than a mechanism of apoptosis resistance. Previous studies have shown that apoptosis-resistance in myofibroblasts it is not necessarily correlated with their organization/quantity of α-SMA (Jelaska and Korn, 2000). Moreover, during wound healing, both myofibroblasts and endothelial cells are eliminated via apoptosis suggesting that the myofibroblast is not intrinsically apoptosis-resistant. It may become resistant under the influence of local factors, as in IPF, or genetic factors, as in our case, which affects the basic mechanisms of apoptotic pathways triggered by a modified environment.

The main changes of the cellular surface during apoptosis were cell shrinkage, the formation of filopodia, blebbing and increased PS expression. Cell shrinkage was the earliest change, starting 30 min after FasL, indicating that apoptotic cell death is coupled to normotonic cell shrinkage, termed apoptotic volume decrease (AVD). AVD has been observed when death receptors, such as CD95, are engaged in human lymphoid (U937), human epithelial (HeLa), mouse neuroblastoma/rat glioma hybrid (NG108-15), and rat phaeochromocytoma (PC12) cells (Okada and Maeno, 2001) and in mouse fibroblasts as demonstrated by the present study. AVD is an early apoptotic process that initially preserves to some extent, the cell's energetic status enabling the apoptotic machinery to work properly (Mills et al., 1999). Our data show that cell shrinkage is not a caspase-dependent process, finding consistent with previous studies (Barros et al., 2002; Moodley et al., 2004). After FasL stimulation, retraction of cellular filopodia was detected after AVD. Notably, the number of filopodia was higher in the Cav-null phenotype, probably reflecting activation status of these cells that coincided with the increased expression of PS on the external leaflet of the plasmalemma proper at 1 h. The externalization of PS peaked at ~3 h and coincided with the initiation of membrane blebbing. It was shown that overexpression of B-cell lymphoma (Bcl-2) proteins completely abolishes membrane blebbing induced by Fas stimulation (extrinsic pathway; Nunez et al., 2010). Based on these findings we assessed its expression in different lung cell populations and found that the expression did not differ between the cell types studied, or between the different lung mouse fibroblasts. These data suggests that Bcl-2 is not an apoptotic molecule in lung fibroblasts. Based on the fact that Bcl-2 overexpression was shown to be involved in rat heart fibroblasts resistance to different apoptotic stimuli (Mayorga et al., 2004), we examined the role of Bcl-2 in apoptotic resistance to FasL by inhibiting its functionality pharmacologically using two Bcl-2 inhibitors. These inhibitors include ABT-263, which inhibits three out of the four members of the Bcl-2 family (Bcl_XL_, Bcl-2, and Bcl-w) and ABT-199, a more selective Bcl-2 inhibitor. The inhibition of Bcl-2 revealed that Bcl-2 is not involved in murine lung fibroblast resistance to FasL-induced apoptosis. The participation of the other two members of this family (MCL1 and BFL1/A), in the response of lung fibroblasts to FasL-induced apoptosis requires further studies and are under scrutiny. Because the expression levels of Bcl-2 and the response of fibroblasts to FasL did not change after its inhibition we concluded that Bcl-2 and at least other members of this family of anti-apoptotic proteins do not participate in mouse lung fibroblast resistance to apoptosis.

This chain of events–AVD, development of filopodia, externalization of PS and blebbing indicates weakening of cytoskeleton-membrane interactions, even if a delayed change in the course of apoptosis at the beginning of apoptotic body formation occurs (Coleman et al., 2001). Almost within the same time frame as the events described above, we detected the presence of cytoskeletal bundles, surfacing of M30 positive lasting for more than 5 h. Their disappearance overlapped, first with nuclear condensation and then with the fragmentation of apoptotic cells into apoptotic bodies. Detection of cleaved K-18 by M30 Ab was evident after 3 h in Cav-null fibroblasts whereas its exposure started after 50 min in wt-fibroblasts. Interestingly, the presence of K-18 in unstimulated fibroblasts was detected in the Cav-null fibroblasts, while its presence in a few fibroblastic cells in wt-mice reflects the presence of myofibroblast. Determining the detailed factor(s)/mechanism(s) triggered by caveolin deficiency responsible for the delayed cytoskeletal bundle formation is under investigation in our laboratory and beyond the scope of this work.

The changes in cellular organelles during FasL-triggered apoptosis were also important, but not as important as the cytoplasmic changes. Significant changes were recorded in the status of mitochondrial continuum of isolated murine fibroblasts after FasL treatment. Mitochondria are dynamic organelles which fuse and divide continuously (Martinou and Youle, 2011), thus their extensive fragmentation (fission) is a constant in apoptosis. Mitochondria integrate death signals through Bcl-2 family members while coordinating caspase activation through apoptogenic factors like cytochrome c. Mitochondrial changes start as alterations in mitochondrial membrane potential that terminate with membrane permeabilization. At the same time, mitochondria fragment and their internal ultrastructure is altered as a result of an activation of the fission machinery and concomitant neutralization of the fusion machinery. The release of tBid, event documented in our isolated fibroblasts, triggers Bax/Bak activation which correlates with a reduction in mitochondrial fusion leading to mitochondrial fragmentation, as indicated by our results. While the association of mitochondria with the endoplasmic reticulum (ER) is important for the transfer of phospholipids and calcium, an extensive mitochondrial fragmentation will enhance these processes and facilitate calcium accumulation in the mitochondrial matrix leading to cell death. The sequestered calcium interacts with cyclophilin D to induce opening of the mitochondrial permeability transition pore (Jeong and Seol, 2008), membrane permeabilization and fragmentation. Our results are suggestive of such events; we are reporting extensive mitochondrial fragmentation and modifications of ER.

Both the Golgi apparatus and the smooth ER were dilated after FasL stimulation. These changes were more dramatic in the Cav-null fibroblasts, particularly in the Cav1^−/−^ fibroblasts. Considering the undeniable evidence for the participation of ER stress in epithelial apoptosis (Kropski et al., 2013), the available data regarding the participation of fibroblast ER in apoptosis are sparse. Even if our data implies that the observed changes in the ER structure found in all of the fibroblast types, are somehow involved in the modification of their apoptotic behavior, more work is necessary to determine the participation of ER stress in caveolin driven fibroblast' fibrosis.

Considering the above characteristic of the apoptotic process, the data provided in this study reveal that fibroblasts isolated from wt- and Cav-null mice share many comparable morphological features of apoptosis that occur over the same time period. However, relevant morphological differences between wt- and Cav-null fibroblasts were noticed: (i) vacuolization in wt-fibroblasts was much more prominent and frequent than in Cav-null fibroblasts, (ii) enhanced filopodia formation as well as increased condensation of the cellular cytoskeleton was evident in the Cav-null fibroblasts, (iii) the presence of α-SMA was 10 times higher in the Cav-null phenotype than in the wt-fibroblasts, suggesting of a constitutively increased number of myofibroblasts, and (iv) a higher percentage of positive cells combined with the delayed formation of M30 antigen in Cav-null fibroblasts. Since our preliminary findings on murine lung fibroblasts exposed to FasL (Predescu et al., 2011, 2012) are substantiated by the work reported here, we may conclude that: (i) wt- and Cav-null fibroblasts undergo sequential cellular changes and share comparable morphological features of apoptosis that occur over the same time period, and (ii) while the Cav-null fibroblasts are more resistant to apoptosis than the wt-fibroblasts, some morphological characteristics such as filopodia formation, delayed cleavage of cytoskeletal bundles and the lack of vacuolization are specific features of Cav-null phenotype resistance to apoptosis.

The loss of mitochondrial membrane potential, observed in the current study, occurred during same time frame as the activation of caspases-8 and the occurrence of Bid fragmentation before caspase-3 activation, while PS expression, cytocheratin-18 cleavage and DNA fragmentation occurred after caspase-3 activation. Thus, in addition to demonstrating that Cav-null fibroblasts are less sensitive to FasL-induced apoptosis than wt-fibroblasts, our data also indicates that several of the morphological features triggered by FasL-induced apoptosis are caspase-dependent.

The way in which cells manage the mode of transition from initiator to execution phase of apoptosis permits their classification in two distinct types, type I and II. In Type I cells the activation of effector caspase is strong and swift, sufficient for robust triggering of death machinery. In Type II cells the activation of initiator caspases is not strong enough for cell death initiation; therefore these cells require a mitochondrial amplification loop for effective activation of apoptosis machinery. They need the presence of the tBid fragment of BH3-only protein Bid generated by activated caspase-8. tBid translocate to the outer mitochondrial membrane (OMM) and allosterically activate Bak. Subsequent oligomerization of Bak and Bax forms pores in OMM and allows escape of proapoptotic protein such as cytochrome c, SMAC/DIABLO etc. The insufficient induction of apoptosis in Type II cells as a failure to activate enough the initiator caspases was demonstrated to be the consequence of low cell surface expression of death receptors (Meng et al., 2011) as CD95, and the presence of caspase inhibitory molecules as XIAP (Jost et al., 2009). Our data revealing: (i) low CD95 surface expression, (ii) high levels of XIAP, (iii) caspase-8 activation, and (iv) the release of tBid, allowed us to concluded that theĭsolated murine lung fibroblasts could and should be classified as Type II cells. Thus, FasL ligation of CD95 triggers the activation of caspase-8 which cleaves the Bid protein to generate the 15 KDa fragment. tBid translocation to the OMM induces Bak/Bax oligomerization and the formation of mitochondrial pore. At this moment the cells release, in the cytoplasm, proapoptotic molecules and reach the point of no return.

In isolated fibroblast we found that the processing of Bid into tBid is primarily facilitated by caspase-8, the first caspase on the Fas apoptotic pathway (Li et al., 1998). Thus, the facilitation of crosstalk between death receptors by activated Bid, and the Bcl-2 controlled apoptotic pathways, amplifies the caspase cascade (Kaufmann et al., 2012) as needed in Type II cells. Our data strongly suggest that this is indeed the case in mouse lung fibroblasts.

When we investigated the molecules involved in the events described above, we found that some pro- and mostly anti-apoptotic molecules were constitutively expressed in wt-fibroblasts and Cav-null fibroblasts. Only Bax was found to be expressed at higher levels in all three of the fibroblasts types. Thus, our data suggests that Bax may participate in the fibrotic process as described in human IPF.

Of the apoptotic regulatory proteins examined, XIAP and cFLIP exhibited the most dramatic changes in expression which were constitutively expressed in fibroblasts (Emblom-Callahan et al., 2010). Previous work based on the expression levels only has suggested the increased presence of XIAP as a possible apoptotic mechanism. Our data support this finding and provide direct functional evidence (siRNA) of its involvement in apoptosis resistance in this cell type.

The mouse fibroblasts express seven out of eight IAPs. The level of XIAP is 2–3 times higher than in other lung cells and 1.5 times higher in the Cav-null fibroblasts when compared to the wt-fibroblasts. The expression of other IAPs (BIRC1, 2, and 3) differed in the mouse fibroblasts, but not significantly. It has been suggested that the variations in survivin (BIRC5) expression is related to the cell cycle; therefore, its direct participation in the apoptotic resistance of fibroblasts as described previously (Sisson et al., 2012) is under scrutiny in our laboratory. Because the down regulation of XIAP increased the sensitivity of fibroblasts to FasL by more than 30%, in all three fibroblast cell types, the apoptosis-resistance of isolated fibroblasts could be explained in part by the constitutively increased expression of XIAP, as long as neither the knockdown of BIRC2 and BIRC3 nor the combined knockdowns with XIAP modifies the cell response to FasL stimulation. Still, in mouse lung fibroblasts with XIAP knockdown, the addition of CHM is needed to induce Fas-mediated apoptosis, albeit at a lower dose (10 μg/mL) than the one used in normal fibroblasts. Our results regarding the direct involvement of XIAP in apoptosis-resistance are in agreement with published data in human fibroblasts (Ajayi et al., 2013) and with its selective expression within the cells (myofibroblasts) of fibroblastic foci and not in the overlapping endothelium (Maher et al., 2010). Our data related to the overexpression of XIAP in lung fibroblasts lead are consistent with published data obtained from T-lymphocytes overexpressing XIAP; BIRC5 protected the cells against FasL-induced apoptosis, even if the protection is trivial (Kaufmann et al., 2012). Thus, our study confirms and extends the contribution of XIAP to mouse lung fibroblasts apoptosis-resistance (our data indicate more than 30% involvement) while demonstrating that other factors are also involved. We found, in preliminary experiments, that the knockdown of DDR1 modifies the fibroblast resistance to FasL only by 11%, as assessed by TUNEL, and thus we continued to search for molecule with a greater impact and found the XIAP and the c-FLICE.

We detected the presence of other “key regulator of apoptosis” -c-FLIP (Ozturk et al., 2012), which was highly expression in all of the mouse lung fibroblast types, with a greater presence in the caveolin null phenotype. Furthermore, we established its participation in fibroblast' apoptosis-resistance via siRNA-mediated knockdown. As in the case of XIAP, down-regulation of c-FLIP increased the sensitivity to FasL-induced apoptosis by 35%. Their double knockdown was additive. The sensitivity of mouse fibroblasts to FasL was around 70%, with very low (<1 μg/mL) amounts of CHM and 80% with low (<5 μg/mL) amounts of CHM needed to induce apoptosis. Of note, even if the increase in apoptotic resistance of mouse lung fibroblasts after the XIAP/c-FLIP double knockdown is significant, the existence of additional mechanisms must explain the remaining 20%. These mechanisms not expected to be vital, and are likely involved in the refinement of the cellular response. c-FLIP overexpression in human lung myofibroblasts from IPF patients has been correlated with their increased resistance to apoptosis (Tanaka et al., 2002; Moodley et al., 2004; Golan-Gerstl et al., 2007); however, a 2010 study (Cha et al., 2010) did not confirm these findings. Therefore, its role remains controversial. We found that its down-regulation did affect the mouse fibroblasts similar to what has been observed in other types of cells (Safa, 2012); c-FLIP increases the resistance to FasL-induced apoptosis through its constitutive overexpression.

Our data demonstrates that tBid, a very effective inducer of mitochondrial pore formation (Ott et al., 2009) coexists with activated caspase-8 and with cFLIP overexpression in the same cells. These findings can be attributed to the action(s) of XIAP, which can directly inhibit caspase-3 and caspase-7 and act at a later stage (caspase inhibition) in the apoptotic pathway.

Based on our results, we are confident that the constitutive overexpression of BIRC5/XIAP and c-FLIP along with their molecular mechanisms represent the primary mechanism of documented mouse lung fibroblast apoptotic resistance.

Our findings regarding the molecular machinery involved in the apoptosis of mouse fibroblasts induced by FasL demonstrated that: (i) there is association between the dynamic succession of morphological changes and commonly used biochemical markers of induced apoptosis, (ii) of all the IAPs, XIAP is the primary contributor through its direct inhibition of executioner caspases, (iii) c-FLIP, as a master regulator of apoptosis, participates in fibroblast apoptosis resistance with the same effectiveness (35%) as XIAP, (iv) caspase-8, and caspase-3 along with caspase-9-albeit to a lesser extent–are the executing molecules, (v) by activating Bid caspase-8 enables the crosstalk between the extrinsic and the intrinsic apoptotic pathways; thus the mouse myofibroblasts from the wt- and Cav-null phenotype could be considered type II cells (Kaufmann et al., 2012) as–they depend on this crosstalk in order for their mitochondria to be affected, (vi) even if the constitutive increase in XIAP-cFLIP is the primary mechanism of fibroblasts resistance to apoptosis, other fine tuning mechanisms (DDR1 and sFas) cannot be disregarded.

Thus, our study extends the existing data by showing that both Cav1^−/−^ (Drab et al., 2001; Razani et al., 2001) and Cav2^−/−^ (Razani et al., 2002) mice have increased deposition of extracellular matrix in the alveolar septa, by substantiating that the main cells responsible are the fibroblasts/myofibroblasts- the cells that do not want to die-, and by providing in depth molecular evidence to explain their constitutive resistance to apoptosis.

In conclusion: (i) the fibroblast is the most apoptosis resistant cell in mouse lungs, (ii) the lack of caveolins accentuates this characteristic via specific biochemical, structural, and phenotypic changes that are similar to those used by the wt fibroblasts but more accentuated in this background, (iii) the structural differences between cav1 null and cav2 null phenotypes (a total lack of cav1 and caveolae in Cav1^−/−^ mice) does not account for the major differences in their response to Fas triggered apoptosis even if signaling via other growth factors and cytokines may differ, and (iv) salient differences between caveolin null fibroblasts underline the adaptive phenotypic capacity for some cellular responses, such as apoptosis resistance, while making it clear that the caveolae are dispensable for a minimal functional adaptability to a certain extent.

## Author contributions

SP and DP conceived and designed the study, analyzed and interpreted the data and edited the manuscript. SP, CB, and DP designed and performed image analyses and edited the preliminary version of the manuscript. JZ, CB, MP, and VG performed cellular and molecular experiments, created the figures and wrote certain sections of the manuscript, helped to design and perform animal cell isolation and characterization experiments. All authors approved the final version of the manuscript.

### Conflict of interest statement

The authors declare that the research was conducted in the absence of any commercial or financial relationships that could be construed as a potential conflict of interest.
