# Supplementary material for: Mouse Lung Fibroblast Resistance to Fas-Mediated Apoptosis Is Dependent on the Baculoviral Inhibitor of Apoptosis Protein 4 and the Cellular FLICE-Inhibitory Protein
Source: Front Physiol. 2017 Mar 14;8:128. doi: 10.3389/fphys.2017.00128 (PMC5348516; doi:10.3389/fphys.2017.00128)
Supplement: Supplementary file 1 [file DataSheet1.PDF]

Supplementary Data

Materials and Methods

Dulbecco's modified Eagle's medium (DMEM), Fetal Calf Serum (FCS), glutamine penicillin/streptomycin, antibiotic-antimycotic solutions, and the -MycoAlert PLUS mycoplasma detection kit were from Lonza (Walkersville, PA). The M30 CytoDEATH Ab and the In situ Cell Death Detection Kit (fluorescein) were from Roche Applied Science (Indianapolis, IN). Dodecyl-sulphate sodium salt (SDS), SDS-PAGE minigels, nitrocellulose membranes, Triton X-100, Nonidet P-40 (NP-40), Tween 20, enhanced chemiluminescence (ECL) detection kit and all chemicals for electrophoresis were from Bio-Rad (Hercules, CA), while the HyBlot CL Film was from Denville Scientific (South Plainfield, NJ). Collagenase type II was from Worthington (Waltham, MA). The following general reagents: Annexin V-FITC, cycloheximide (CHM), propidium iodide, pan caspase inhibitor (CI) Z-VAD-FMK and 7-amino actinomycin D were from Sigma-Aldrich (St Louis, MI), the Bcl2 inhibitors ABT-199 and ABT-263 were from Santa Cruz Biotechnology (Dallas, TX). The recombinant active caspase-3, -8 and -9 along with their specific substrates: DEVD-pNA for caspase-3, IETD-pNA for caspase-8, and LEHD-pNA for caspase-9 were from Enzo life science (Farmingdale, NY). Caspase-3 peptide inhibitor Q-DEVD-OPh (*N*-(2-Quinolyl)-Asp(OMe)-Glu(OMe)-Val-Asp(OMe)-(2,6-difluorophenoxy)-methyl ketone), caspase-8 inhibitor Q-IETD-OPh (*N*-(2-Quinolyl)-Ile-Glu (OMe)-Thr-Asp (OMe)-(2,6-difluorophenoxy)-methyl ketone) and caspase 9 inhibitor Q-LEHD-OPh (*N*-(2-Quinolyl)-Leu-Glu(OMe)-His-Asp(OMe)-(2,6-difluorophenoxy)-methyl ketone, the BrdU Immunostaining kit ab125306, anti Thy-1 and Anti-Reticular Fibroblasts and Reticular Fibers [ER-TR7] antibodies were from Abcam (Cambridge, MA).

We used the following antibodies (Abs): goat anti-PHD4/prolyl hydroxylase and rabbit anti-CD95 Abs from Abcam; anti-CD95 mAb (APO1-3) used for cell ELISA from Axxora; rabbit and goat Abs recognizing mouse Baculoviral IAP Repeat-Containing Proteins (BIRC) 1-6, Bad, Bax, Bak, Bid, Bcl-2, Bcl_{XL}, Discoidin Domain Receptor 1 (DDR1), cav1 and cav2, anti-CD 68, anti-CD 45, anti-vimentin, anti-factor VIII, anti-CD31, anti-podoplanin, anti α-smooth muscle actin (α-SMA), anti β-actin from Santa Cruz Biotechnology (Dallas, TX). The rabbit anti

BIRC7 was from Novus Biologicals (Littleton, CO), the Rat anti cleaved Bid was from R&D Systems Inc. (Minneapolis, MN), and anti-cellular c-FLIP and anti-total caspase-8 were from BD Bioscience (San Hose, CA). Goat anti-mouse and anti-rabbit conjugated to Alexa Fluor® Plus 488, or Alexa Fluor® Plus 555 were used as reporters for fluorescent immunostaining, while Goat anti-mouse and anti-rabbit Horse Radis Peroxidase conjugated were used for WB and ELISA.

The following kits were used: ELISA kit (DY435) for detection of mouse Fas, the kit (MFL00) for detection of sFas and the recombinant mouse Fas Ligand/TNFSF6 protein from R&D Systems Inc. (Minneapolis, MN). JC-1 Mitochondrial Membrane Potential Assay Kit was from Cayman Chemical Company (Ann Arbor, MI). Wizard® Genomic DNA Purification Kit (A1120) from Promega (Madison, WI). Caspase Colorimetric Protease Assay Sampler Kit KHZ1001from Invitrogen, while Caspase-3 Cellular Activity and Colorimetric Activity Assays kits for caspase-8 and -9 from Chemicon International (Temecula, CA). 3-(4,5-Dimethylthiazol-2-yl)-2,5-diphenyltetrazolium bromide (MTT) Cell Proliferation Assay kit from ATCC (Manassas, VA). RNeasy MiniRNA isolation kit from Qiagen (Valencia, CA). The recombinant active caspase-3, -8 and -9 along with their specific substrates: DEVD-pNA for caspase-3, IETD-pNA for caspase-8, and LEHD-pNA for caspase-9 were from Enzo life science (Farmingdale, NY).

Animals

Mice were housed under standardized pathogen-free conditions in the Rush University animal facility. All surgical procedures were done under anesthesia using a mixture of ketamine (60 mg/kg), and acepromazine + xylasine (2.5 mg/kg + 2.5 mg/kg), in 0.05mL PBS provided by the Rush animal facility. The $Cav1^{-/-}$ and $Ca2^{-/-}$ mice were genotyped every 3-6 months and breed to form Cav-null colonies.

Cell isolation, culture, treatment and fractionation

For the isolation and culture of mouse fibroblasts, sterile removed lungs were flushed blood free using DMEM + 2 times the concentration of pen/streptomycin antibiotics, then transferred to a sterile hood and cleaned of trachea, large bronchia and all large vessels. The parenchyma was minced into ~3mm^3 pieces, washed briefly with sterile medium and distributed

> 30 pieces / 100mm Petri dishes that were transferred at 37^0C and 5% (v/v) CO^2 in complete medium. They were untouched for 96h after which the medium was changed, and the culture was monitored until outgrowth from the tissue pieces was noticed (~7days). The tissue pieces were removed, and the media was changed every third day until confluence was reached. Mouse fibroblasts, obtained from a pool of 4-6 lungs from each type of mouse, were cultured in T75 flasks using DMEM low-glucose (1000 mg/L) supplemented with 10% FCS, and penicillin /streptomycin antibiotic solution. The fibroblasts cultures were maintained in DMEM + 10% FCS, that was changed every third day and used between passages 4-8. The purity of established fibroblasts cell lines was checked by staining with anti-vimentin, anti-collagen type I, III and IV, anti-CD45 (pan-leukocytes), -CD 68 (macrophage specific), -factor VIII antigen and -CD31, (endothelial specific), - podoplanin (lymphatic endothelia specific), and α-SMA Abs.

All established cell cultures: fibroblasts, epithelia and endothelia were periodically (every three months) tested for mycoplasma presence using a kit from Lonza.

Induction of apoptosis

For all experiments, the fibroblasts were made quiescent by growing them in DMEM + 0.1%FCS for 24 h and preliminary experiments carried out with different concentrations of FasL (5, 10, 30, 60, 90, 100 µg/mL) and CHM (10, 20, 40, 60, 90, 100, 120 µg/mL) allowed us to establish the cellular threshold - 90 µg/mL FasL in the presence of 100 µg/mL CHM - needed for apoptosis induction, this condition will be referred through the paper as FasL-induced apoptosis. Additionally, isolated fibroblasts were treated for different time points (1, 2, 4, 8, 12, 24 and 48h); and also we changed the succession of FasL and CHM administration in order to establish the best conditions for apoptosis induction.

siRNA Studies

Transfection with individual siRNA for XIAP, c-IAP1, c-IAP2 and c-FLIP or combinations of two siRNA for double knockdown, was carried out using Silencer Select siRNAs (Life technologies, Grand Island, NY), validated for BIRC2, BIRC3, BIRC4 and c-FLIP. For this condition, cells 80-90% confluent, were washed in medium without serum, trypsinized and adjusted to 5×10^6 cells/mL in RPMI 1640 supplemented with 10% fetal bovine serum, then electroporated by single pulse electroporation in a Gene Pulser® Cuvette (Bio-Rad)

with a 0.4 cm electrode gap using a Gene Pulser Xcell™ Electroporation system (Bio-Rad, Hercules, CA) set to 230 volts, 875. In the experiments involving double transfections the cells were used after 48-72h post-second transfection. siGENOME SMART pool siRNA and Non-targeting siRNA pool (from Thermo Scientific) along with *Silencer* Negative Control #1 siRNA for single transfections, and *Silencer* Select Negative Control #2 siRNA (from Life Technologies) for double knockdown experiments, were included in each experiment.

Measurement of CD95, FasL bound to cell surface and secreted into cell culture media

The quantity of CD95 expressed in the surface of isolated fibroblasts was measured using a cell-Elisa assay. Confluent cells at passage 3, were detached with trypsin (0.25% for 2-3 min), re-suspended in cold PBS, washed by centrifugation (2 x 5 min at 800g) in an Eppendorf centrifuge and after the final wash re-suspended in culture media at a density of 10^5 cells/mL. Same number of cells were plated/well in a six well plate in 3 mL media/well and allowed to attach for 24h. In preliminary experiments we found that 1 to $5x10^5$ per well are needed for optimal detection of CD95 in the isolated fibroblasts membranes. Next the cells were washed with culture media without FCS and cultivated another 24h in culture media containing 0.5% FCS. The media was changed with 1 mL of fresh media over which an equal amount of freshly prepared 8% paraformaldehyde was added and incubate at RT for 15 min. The fixative solution was aspirated gently and the fixed cells were washed 3 times briefly (2 min) with excess PBS (6 mL/well). The PBS was removed, and the wells were incubated with 2X blocking solution (5% casein in PBS at 1 ml/well) for 2h at RT. After the removal of blocking solution the cells were incubated with the anti-CD95 mAb diluted 1:500 in the same blocking solution (500µl/well) overnight at 4^0C. After the removal of primary Ab solution the wells were washed (3x5 min) with washing buffer and then with 500µl/well secondary Ab (anti-mouse IgG-HRP coupled from KPL) diluted 1:1000 in blocking buffer diluted 1:10. The well were incubated for 2h at RT in the Ab solution, then washed (5x2 min in PBS) and after the final wash the reacted cells were incubated with the HRP Development Solution substrate (KPL) and the absorption of developing color was recorded after 5 min as an endpoint data at 650nm in an Epoch plate reader. The data were exported in Excel that was used to analyze the raw data and to create the ensuing plots. We used a plate shaker (~300 rpm) during the incubation steps; after all incubation steps: blocking,

first and secondary Abs and any step involving removal of buffer or solution, the plate was gently blotted upside down on a paper towel.

The relative amounts of FasL bound to cell membrane was obtained by WB as follows: the crude membrane fraction was solubilized in an extraction buffer containing: 50 mM Tris-HCl, pH 7.4 containing 150 mM NaCl, 1 mM Na2EDTA, 1% (v/v) Nonidet P-40, 0.1% (w/v), SDS, 1% (w/v), sodium deoxycholate, 20 mM α-glycerophosphate, 1mM Na3VO4, 1 mg/ml leupeptin, 2 mg/ml aprotinin, and 1 mM PMSF.

Quantitative PCR (qPCR) for FasL mRNA was performed with the following primers: F: 5'-GTTCTGGTTGCCTTGGTAGG-3'; R: 5'-GACCAGAGAGAGCTCAGATACG-3' which generate a 534-bp fragment of FasL. For each condition, total RNA was extracted from $2x10^5$ fibroblasts using the RNeasy kit, as per manufacturer recommendations, and then reverse transcribed and amplified by PCR using the following conditions: 1 cycle at 94°C for 5 min, then 35 cycles at 95°C for 40 seconds, 58°C for 1 min, and 72°C for 1.5 min. The PCR products were electrophoresed in a 2% agarose gel, using a plasmid containing the human FasL cDNA as a positive control.

AnnexinV staining

We monitor the expression of PS on $1x\ 10^5$ cells seeded on cover-slips. The next day, the adherent cell layer, was treated with FasL in presence of CHM for the times mentioned in figure legends, then washed 3x5 min with HEPES+ 0.1% BSA, followed by 3 washes for 3x5 min with HEPES only. The samples were incubated with labeling solution (HEPES buffer containing AnnexinV-FITC and propidium iodide) for 30 min.

TUNEL and cellular DNA fragmentation

Sub-confluent cells (80-90% confluent), on coverslips, were exposed to FasL and washed 3 x 2 min each with cold PBS, then fixed with 4% paraformaldehyde in PBS, prepared from powder, washed again with PBS and permeabilized with 0.1% Triton X-100 in 0.1% sodium citrate, for 2 min on ice. For immunostaining, the TUNEL mixture, prepared as per manufacturer protocol, was applied to permeabilized cells for 30 min at 37^oC, then washed twice with PBS, mounted with Prolong-antifade media containing DAPI, and examined with a

Zeiss Axioimager M1 fluorescence microscope using a 488nm wavelength for excitation and 550nm for detection.

Caspase activation

5 x10^6 cells (wt-, Cav$1^{-/-}$, Cav$2^{-/-}$ fibroblasts) per sample treated with FasL + CHM were re-suspended in 50 µL of ice-chilled lysis buffer (KHZ1001-kit), and we followed the protocol recommended by the manufacturer. The plates were read at 405nm in an Epoch reader. All experiments were performed in triplicates and the fold-increase in caspase-2, -3, -6, -8, and -9 were determined by comparison to non-treated controls.

Using the protocols provided by the corresponding kits, 2 x10^7 cells, for every type of fibroblasts, were stimulated with FasL and used to measure the increase in enzymatic activities of caspase-3, -8, -9, at 24 h and 48h. A standard curve was generated for every caspase using purified enzymes, the caspase buffer: 50 mM Hepes, pH 7.2, 50 mM NaCl, 0.1% Chaps, 10 mM EDTA, 5% Glycerol, and 10 mM DTT and p-nitroaniline at 50µM concentration, while the conversion factor for the specific substrate was used to calculate enzyme activity as pmol/min. The results expressed as pmol/min/µg protein are presented as fold increase in different caspases activity by comparison with fibroblasts not treated with FasL.

Mitochondrial Function

The mitochondrial transmembrane potential ($\Delta\Psi m$) was determined with the JC-1 assay kit as per manufacturer instructions using a 96 well black plate, in which 5 x 10^5 cells were seeded, allowed to adhere for 24h, then treated with FasL + CHM. JC-1 dye forms bright red aggregates in intact mitochondria, while it remains as a green monomer in apoptotic organelle. JC-1 dye forms bright red aggregates in intact mitochondria, while it remains as a green monomer in apoptotic organelle. The fluorescence of JC-1 was analyzed using the EPOCH plate reader, first at 560 and 595 nm for the detection of J-aggregates and then at 485 and 535 nm for the detection of JC-1 monomers. The ratio of the fluorescence intensity J-aggregates/monomers was used to calculate changes in $\Delta\Psi m$ as induced by apoptotic treatment.

The structural integrity of the mitochondrial continuum was assessed by transmission electron microscopy (TEM) of unstimulated and FasL-stimulated fibroblasts, as well as by determining its DNA copy number after FasL induced apoptosis of mouse fibroblasts using the

method of (Miller et al., 2003) and a 211bp fragment of the mtDNA 12S RNA gene as a probe**.** The Wizard® Genomic DNA Purification Kit was used to extract the fibroblasts' genomic DNA; the pGEMTE-12S vector containing a 1603 base pair (bp) fragment of mitochondrial transcription factor A gene was first amplified and a 1603 bp fragment was cloned into pGEM-T vector from Promega as in (Andrews et al., 1999) to yield pGEMTE-12S vector. This vector was used as an internal control in the qPCR reaction. Primers for qPCR were from Qiagen (PPM26131A) and 30 cycles of amplification were used.

Keratin 18 fragmentation assay

Keratin 18 (K18) as a type I intermediate filament protein and one of the major component of epithelial cells, is cleaved by caspases, exposing a neo-epitope (M30) that is specifically recognized by M30 CytoDEATH™ monoclonal Ab, which detects only apoptotic cells (not viable or necrotic cells). Thus we have used it for immunostaining of isolated fibroblasts from wt, Cav1 and Cav2 $^{-/-}$ mice as per manufacturer reference. Briefly: the lyophilized Ab was reconstituted in 550 µL double distilled water and from this stock solution a dilution 1:50 was made immediately before use in PBS +1% BSA +0.1% Tween 20. The growing media was removed and the coverslips with confluent fibroblasts were washed 3x10 seconds in PBS and then fixed in ice cold methanol at -20^{0}C for 15min. The fixed cells were washed with PBS + 0.1% Tween 20 3x5 min and then overlaid–cell layer down - on top of a drop of 75µl of diluted anti-M30 Ab solution for 2 h at 20-25^{0}C. After washing as above (3x5 min washing buffer) the immunostained coverslips were incubated with a fluorescently labeled anti-mouse IgG (10 µg/mL) for 1h at RT. Finally the stained coverslips were washed twice for 5 min with the same washing buffer and mounted (with) and viewed using the AxioPlan microscope and the appropriate filters for the fluorophore.

Western blotting and immunofluorescence

Total protein, 20 – 80 µg per lane, were run at 150V on an SDS-PAGE minigel, the separated bands transferred to nitrocellulose membranes (NC), and strips of NC were incubated with the primary Abs and processed as follows. After 1h blocking in PBS + 5% blotto the membranes were incubated with primary Ab (diluted to the necessary concentrations in the same blocking buffer) for 2h at RT. The strips were washed 3x15 min in PBS + 0.3% Tween 20 + 0.05% NP40

and then incubated with the reporter Abs diluted in PBS + 0.1% blotto for 1h at RT. After washing 3x10 min with PBS + 0.3% Tween 20 the reaction was visualized using the ECL kit from Thermo Fischer as detailed in the accompanying instructions and the HyBlot CL film for exposure. When needed, the films were used for semi-quantitative assessment of bands intensity using the available Image J software.

For immunofluorescence**,** sub-confluent monolayers of fibroblasts were washed with ice-cold PBS, fixed/permeabilized with methanol (5min, -20°C), blocked with 1% BSA in PBS (PBS-BSA) for 1h at RT, and then incubated with the primary Abs (diluted in PBS-BSA) for 1-2h at RT, washed with 0.1% BSA in PBS, incubated with the corresponding secondary Abs (diluted in PBS-BSA) tagged with Alexa Fluor 488 or Alexa Fluor 549, (1h, RT), washed again, mounted with Prolong antifade reagent, and examined with a Zeiss Axioimager M1 fluorescence microscope. All experiments involving Abs were repeated 4 times and controls with species-specific and isotype-specific immunoglobulin G were performed in preliminary experiments and repeated 3 times for each condition.

Transmission Electron Microscopy (TEM) and Scanning Electron Microscopy (SEM)

For TEM, wt-, Cav 1$^{-/-}$ and Cav 2$^{-/-}$ fibroblasts were grown in 35 mm plastic Petri dishes until 70-80% confluent, exposed to FasL, then at predetermined time point, the media was removed and the adherent cells were prepared for flat embedding as in (Predescu et al., 2003). Briefly, the monolayers were rinsed with 0.1M cacodylate buffer, pH 7.4 and fixed with 2% glutaraldehyde in 0.1M cacodylate buffer + 5% sucrose for 30 min at RT, rinsed again 6 times (5 min each) with 0.1 M sodium cacodylate buffer pH 7.4 and post-fixed with reduced osmium (1% OsO_4 + 1% potassium ferrocyanide in 0.1M Sodium cacodylate, pH 7.4) for another 30 min, then briefly washed (4 x 1 min) washed with distilled water and en-block stained with 5% Mg uranyl-acetate overnight, at RT. The fixed monolayers were washed (3 x 1 min) with distilled water, dehydrated for 5 min in increased concentrations of ethanol (50%, 70%, 80%, 95%), and then placed in 100% ethanol (200 proof) 3 x 15 min each. The membranes were then infiltrated for 1h with a mixture of 1:1 (v/v) absolute ethanol: Epon 812, then for another hour with Epon 812 and finally the Petri dishes were filled with Epon 812 and let to cure overnight, under vacuum at RT. In the next day they were moved into an oven at 65^{0}C, for 48h. Blocks of ~ 10^{x6} mm, were cut from

polymerized resin (the plastic bottom of the dish included), then ultrathin sections (~60 nm thick) were cut from small pyramids centered on the interface between resin and plastic and mounted onto copper grids. Stained sections (lead citrate and uranyl acetate), were examined with a Jeol 1220 TEM at 80kV, and images acquired with a Gatan camera.

The SEM examination of cultured fibroblasts was performed as in (Knezevic et al., 2009). Briefly, cells were grown on polylysine-coated glass coverslips, rinsed with PBS, fixed with 2.5% glutaraldehyde in PHEM buffer (15 min, RT), submerged in 1% tannic acid in 0.1 mM cacodylate buffer, pH 7.2, overnight, post-fixed in 1% OsO_4 in 0.3 mM sodium cacodylate for 15 min, washed with water for 5 min, dehydrated through increasing concentrations of ethanol, and exchanged to pure hexamethyldisilazane. The fixed cells were coated with a 4-nm layer of a platinum-carbon mixture using a Cressington sputter coater, observed at 15-20 kV and micro-graphed using a Jeol SM 6320F field emission scanning EM.

Protein expression levels of different caspases in isolated mouse lungs fibroblasts†

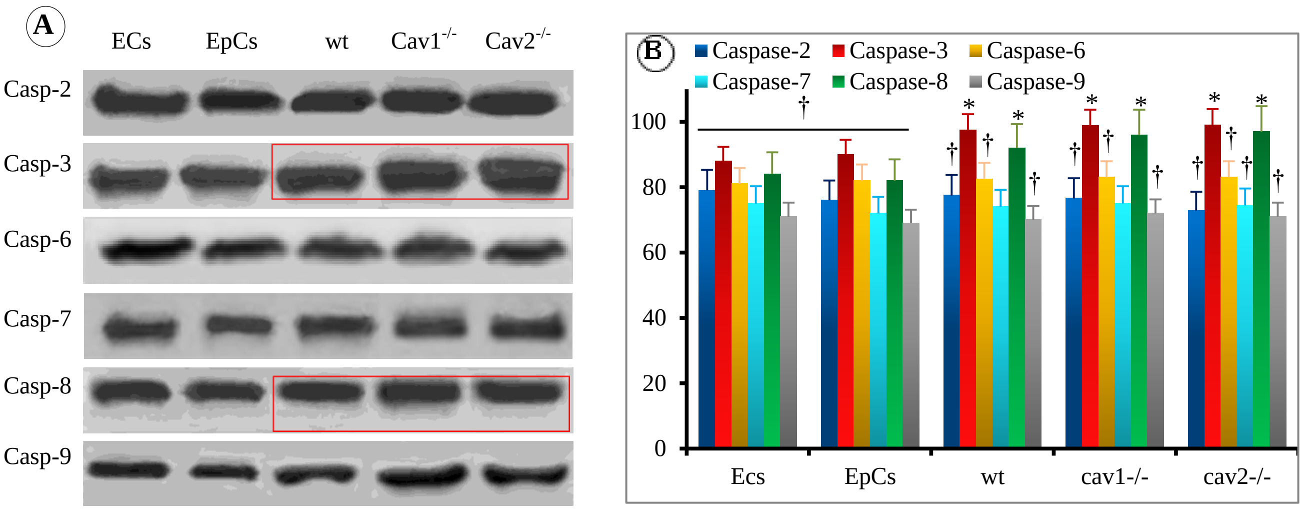

(A) WB analyses for caspase protein expression in the isolated fibroblasts demonstrating the presence of caspase-2, caspase-3, caspase-6, caspase-7, caspase-8 and caspase-9 in all types of fibroblasts isolated from mouse lungs, as well as in the ECs and EpCs; n = 5 for every caspase. Are highlighting the constitutive increase in the levels of expression for caspase-3 and caspase-3 in all types of isolated fibroblasts. (B) Relative quantitation of blots shown in A demonstrate that in the isolated fibroblasts caspase 3 and caspase 8 levels differ significantly (*****p < 0.003) when compared with isolated ECs and EpCs. The Caspase-2, Caspase-6 and Caspase-7 expression did not revealed differences between different types of cells (p < 0.5) or between different types of isolated fibroblasts (†p < 0.41).

Figure 1S

General proteolytic activity of identified caspases in isolated and untreated fibroblasts (U) and in fibroblasts exposed to FasL.

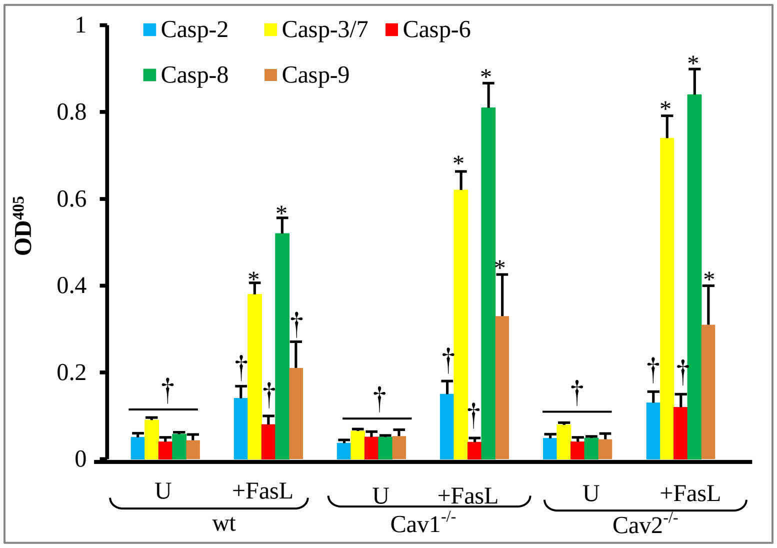

The identified caspases display proteolytic activity in the wt-fibroblasts and the Cav-null fibroblasts. While low proteolytic activity was present for all identified caspases in the absence of any stimulation, 24h after FasL treatment, the activity of caspase-8 (green bars), caspase-3 (yellow bars), and caspase-9 (brown bars) was noticeably higher; n = 6 for every cell type and condition; $^*p < 0.05$ when FasL-stimulated are compared with untreated (U) cells and $^\dagger p < 0.3$ in between all the isolated cells.

Figure S2

Relative amounts of XIAP and cFLICE in different mouse lung cell types

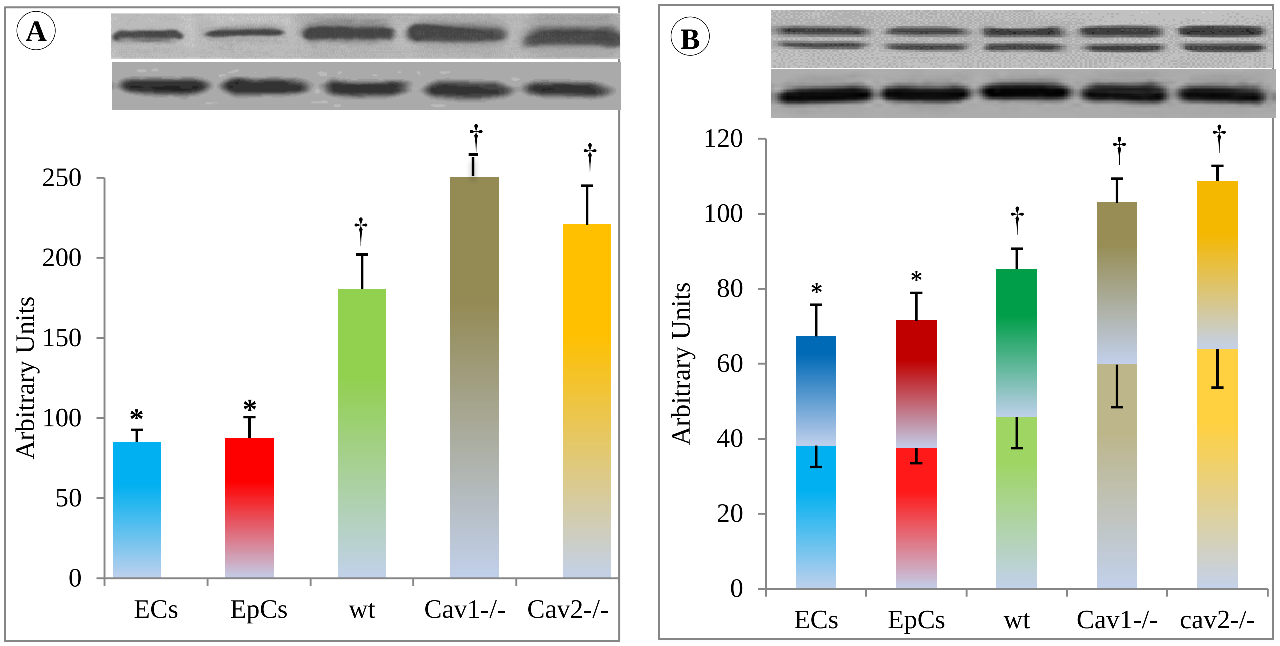

Immunostaining for XIAP (A, upper panel) and for cFLICE (B, upper panel) showing variances in their expression between different types of cells isolated from mouse lung. Anti β-actin Ab was used on or striped and re-probed blots from upper panel as loading control (A and B lower panels). **(A)** Note the augmented expression of XIAP in isolated lung fibroblasts (2 to 3 times more) when compared to ECs and to EpCs, while no differences between ECs and EpCs are detected. n = 6 for each condition and each type of cell;$^{*}p < 0.22$ and $^{\dagger}p < 0.001$. **(B)** The expression levels of cFLICE showed the same trend as in the case of XIAP. Largely, the levels of cFLICE are lower than the levels of XIAP, they are 40-50% higher in the isolated Cav-null fibroblasts than in ECs and EpCs and we found no differences in its expression between ECs and EpCs. n = 6 for each condition and each cell type;$^{*}p < 0.36$ and $^{\dagger}p < 0.002$.

Figure 3 S
